# Dermatophytosis: An Update on Global Epidemiology

**DOI:** 10.3390/jof12070503

**Published:** 2026-07-09

**Authors:** Laura Beatriz Borim da Silva, Laríssa Santos de Oliveira, Thiago Blanco Parra Furlan, Bruna Carolina Teixeira Almeida, Maíra Terra Garcia, Yinggai Song, Nalu Teixeira de Aguiar Peres, Paulo Henrique Fonseca do Carmo

**Affiliations:** 1 Departamento de Genética, Microbiologia e Imunologia, Instituto de Biociências de Botucatu, Universidade Estadual Paulista (UNESP), Botucatu 18618-689, SP, Brazil; 2 Departamento de Microbiologia, Instituto de Ciências Biológicas, Universidade Federal de Minas Gerais (UFMG), Belo Horizonte 31270-901, MG, Brazil; 3 Departamento de Biociências e Diagnóstico Bucal, Instituto de Ciência e Tecnologia, Universidade Estadual Paulista (UNESP), São José dos Campos 12245-000, SP, Brazil; 4 Department of Dermatology and Venerology, Peking University First Hospital, Beijing 100191, China; 5 National Institute of Science and Technology in Human Pathogenic Fungi, São Paulo 13083-862, SP, Brazil

**Keywords:** dermatophyte, prevalence, vigilance, antifungal resistance

## Abstract

Dermatophytosis is a cutaneous mycosis caused by keratinolytic fungi, classified as dermatophytes, affecting 20–25% of the global population and representing a significant public health concern. The disease manifests as circular, erythematous, pruritic, and desquamative skin lesions, hair breakage and loss, and nail degradation, leading to considerable morbidity. In addition, dermatophytosis can markedly impair patients’ quality of life. Despite the high global prevalence of dermatophytosis, the causative agents are often misdiagnosed, and there is limited data on the epidemiology and genomic surveillance of dermatophytosis worldwide. This review aims to update the global epidemiology of dermatophytosis and dermatophytes while addressing their taxonomy, pathogenesis, virulence factors, clinical manifestations, and antifungal therapy. Consistently, *Trichophyton* spp., particularly *T. rubrum*, *T. mentagrophytes,* and *T. interdigitale*, remain the predominant pathogens worldwide. Recently, *T. indotineae* has gained prominence due to its global dissemination, significant terbinafine resistance, extensive lesions, therapeutic failure, and recurrence. Furthermore, studies have reported endogenous cases of *T. indotineae* infections in Asia and Europe, and exogenous reports in the Americas and Oceania. Among non-*Trichophyton* dermatophytes, *Microsporum canis* and *M. audouinii* stand out as relevant pathogens, particularly in endemic regions and specific clinical settings, such as scalp infections. Overall, these factors emphasize the importance of global vigilance regarding dermatophyte dissemination. This review also highlights a discrepancy between accurate fungal identification and global reporting, raising concerns regarding epidemiological surveillance and underscoring the need for improved strategies to manage dermatophytosis worldwide.

## 1. Introduction

Dermatophytosis is one of the most prevalent cutaneous mycoses worldwide, affecting approximately 25% of the global population and posing a significant public health concern [1]. These infections are caused by keratinophilic filamentous fungi known as dermatophytes. These ubiquitous pathogens produce enzymes capable of metabolizing the keratin present in human and animal tissues, such as skin, nails, and hair [2,3]. Characteristic clinical manifestations include circular, erythematous, pruritic, and desquamative skin lesions, hair breakage and loss, and nail degradation [4]. Dermatophytosis can significantly impair patients’ quality of life, often associated with depression and social awkwardness [5]. Although the clinical features are often typical, the causative agents are frequently misdiagnosed due to limitations in diagnostic methods. Misdiagnosis impairs epidemiological data and hinders the effective management of infections worldwide, particularly in endemic regions. Consequently, the prevalence of dermatophytosis is often underestimated, diminishing recognition of its impact and the global dissemination of dermatophytes, as has been observed with the *Trichophyton indotineae* and other resistant isolates [6,7,8]. In this context, this review aims to provide an updated overview of the taxonomy, pathogenesis, virulence factors, clinical manifestations, and antifungal therapy of dermatophytosis, with a particular focus on the global epidemiology of dermatophytes.

## 2. Dermatophytes

Dermatophytes are hyaline filamentous fungi that share the common characteristic of being able to degrade and use keratin as a sole nutrient source. Taxonomic classification of dermatophytes has recently undergone revision due to advances in fungal identification using molecular techniques. Traditionally, these fungi were grouped into the genera *Epidermophyton*, *Microsporum*, and *Trichophyton* based on in vitro morphology, focusing only on the anamorphic stages, and clinical features such as the appearance and location of the lesions [9], often leading to misdiagnosis. However, with the incorporation of phylogenetic analyses based on the conserved internal transcribed spacer (ITS) region and the β-tubulin gene, as well as considering the teleomorphic stage, taxonomic studies have proposed that the dermatophyte group includes the genera *Trichophyton*, *Nannizzia*, *Microsporum*, *Lophophyton*, *Arthroderma*, *Ctenomyces*, *Guarromyces*, and *Paraphyton*. In this context, *Epidermophyton* is considered as an independent genus when ITS analysis is used alone, but is included within *Nannizzia* when β-tubulin data are also considered [10,11,12].

In addition to their taxonomic classification, dermatophytes can also be categorized according to their ecological niche as anthropophilic, zoophilic, or geophilic. Anthropophilic species include *Epidermophyton floccosum*, *Microsporum audouinii*, *Trichophyton interdigitale* (formerly *T. mentagrophytes* var. *interdigitale*), *Trichophyton rubrum*, and *Trichophyton tonsurans*. Particularly, *T. rubrum* accounts for 50% to 90% of the reported cases of dermatophytosis [4]. Zoophilic species include *Microsporum canis*, *Microsporum gallinae*, *Trichophyton equinum*, *Trichophyton mentagrophytes* (formerly *T. mentagrophytes* var. *mentagrophytes*), and *Trichophyton verrucosum*, whereas geophilic species include *Microsporum boullardii*, *Microsporum cookei*, *Nannizzia gypsea* (formerly *Microsporum gypseum)*, and *Microsporum nanum* [13]. Anthropophilic species are often associated with chronic dermatophytosis, characterized by a mild inflammatory response and increased therapeutic challenges, reflecting their adaptation to the human host [13,14,15]. In contrast, zoophilic and geophilic species tend to elicit more intense inflammatory reactions, which may be misdiagnosed as bacterial infections or non-infectious dermatitis, posing a diagnostic challenge [16]. Therefore, understanding both taxonomy and ecology is clinically relevant, as it informs transmission patterns, inflammatory responses, and therapeutic decisions [17,18].

### 2.1. Pathogenicity Factors of Dermatophytes

Dermatophytes can establish and colonize the host tissue without prior trauma, spreading through direct and indirect contact. Fungal cells can remain viable for extended periods in desquamated skin cells, hair, and nails, as well as in the environment on objects, such as manicure appliances, towels, and hairbrushes, for over six months, which contributes to their high prevalence [9]. Consequently, these infections have been mistakenly considered sexually transmitted diseases [19].

The initial phase of the infection involves adhesion, mediated by conidial cell wall adhesins that promote attachment to keratinized tissues. In species such as *T. mentagrophytes*, conidia can project fibrillar structures that enhance adhesion [4]. These adhesins bind to host surface molecules, such as mannose and galactose, enabling the initial attachment, colonization, and tissue penetration. Rapid adhesion is crucial for the fungus to resist being removed by skin desquamation and keratinization. This enables dissemination throughout the skin, leading to increased lesion areas or even invasion of deeper layers [15]. Dermatophytes scavenge for nutrients in the host tissue and secrete a broad variety of enzymes to break down macromolecules, which are then transported into the fungal cell and metabolized. The main enzymes display keratinolytic properties, i.e., keratinases, which degrade the host keratin providing essential nutrients for fungal growth [20]. Among these, exo- and endoproteases, such as subtilisins (Sub) and metalloproteases (Mep), are closely linked with fungal virulence and infection severity [4]. Mep4, Mep5, and Sub6 have been evaluated in *T. mentagrophytes*, Sub3, Sub4, and Mep4 in *T. rubrum*, and Sub3 and Sub6 in *Arthroderma benhamiae*. Notably, Sub6 has recently been considered non-essential for pathogenicity, but it has the potential to be an infection marker, and therefore, enhancing diagnostic tools [4,21,22]. Although not yet well validated for clinical diagnosis, experimental studies have identified *SUB* genes and Sub endoproteases as potential diagnostic markers through various techniques, including polymerase chain reaction (PCR) [23], two stage multiplex PCR [24], proteomic analysis [25], and anti-peptide antibodies with a catalyzed reporter detection system (immune-CARD) [26]. These approaches achieved positivity rates ranging from 66% to 100% using animal, human, and soil samples containing different dermatophyte species. In addition, leucine aminopeptidases (Lap1 and Lap2), and dipeptidyl peptidases (DppIV and DppV) are also present in *T. rubrum*, *Trichophyton violaceum*, and *Trichophyton benhamiae*, highlighting the diversity of proteases involved in adaptation and virulence, and therefore allowing the colonization of a diversity of ecological niches [4].

Extracellular keratin degradation begins with proteases, but also depends on sulfite secretion, which increases substrate accessibility. Due to its high cysteine content and disulfide bonds, keratin resists proteolysis, requiring sulfitolysis to enable enzymatic degradation [27]. Once the keratin structure has been disrupted, proteases complete its degradation and the resulting amino acids, including cysteine, are internalized by the fungus [4]. Cysteine is then metabolized by the cysteine dioxygenase (Cdo1), contributing to sulfite production. Then, sulfite is exported to the extracellular environment via the sulfite efflux pump Ssu1, thus sustaining the keratin degradation process [27]. It is notable that the genes encoding *CDO1* and *SSU1* have been identified as important virulence factors in dermatophytes, as *A. benhamiae* knockout mutants show impaired growth when cultured on human hair and nails [3,28].

The regulation of these enzymes is closely tied to the ability of dermatophytes to respond to the host environment. Through transcription factors such as PacC, fungi adjust protein expression according to local conditions, including pH variations. The skin is normally acidic, but becomes more alkaline at infection sites, favoring keratinase production [9,15,20]. Optimal fungal growth occurs at temperatures below body temperature, from 27 °C to 33 °C. Therefore, proper protein folding and stability, mediated by heat shock proteins (Hsps), are essential for coping with host-induced stress. For instance, Hsp90 in *T. rubrum* has been shown to play a crucial role in keratin degradation, contributing to environmental adaptation and maintenance of virulence [29,30]. In addition, transcription factors such as HacA, StuA and Ap1 in *T. rubrum* regulate a myriad of cellular events, including the regulation of keratinases and metabolic changes during keratin degradation, as well as cell wall remodeling. These changes also modulate the hosts’ immune responses, therefore contributing to dermatophytes pathogenicity [31,32,33].

Beyond proteases, dermatophytes employ other strategies to establish infection. Cell wall components such as mannans have anti-inflammatory properties, inhibit keratinocyte proliferation and prevent epidermal shedding, thereby contributing to chronicity in anthropophilic species [15]. At the host’s physical barrier, these fungi also produce toxins, such as xanthomegnin, released by *Trichophyton megninii*, *T. rubrum*, and *T. violaceum*, and hemolysins, produced by *T. rubrum* and *T. interdigitale*, contributing to tissue damage [34].

Once the keratinocyte barrier has been overcome, dermatophytes trigger the recruitment of neutrophils and other leukocytes. These cells release pro-inflammatory cytokines and antimicrobial peptides, thereby initiating inflammation [35]. The intensity and profile of this immune response vary according to the species involved, and anthropophilic dermatophytes induces lower cytokine production, resulting in milder inflammation [15]. This modulation is influenced by fungal components, including secondary metabolites, that interact with host receptors and influence the activation of different lymphocyte subtypes (Th1, Th17, Th2, and Treg), determining whether infection proceeds acutely or chronically [34]. Furthermore, some dermatophytes can survive the microbicidal activity of phagocytes by germinating into hyphae within macrophages, disrupting the membrane, causing cell death [35]. To persist within the host, dermatophytes can also form biofilms with a dense extracellular matrix composed of fungal cells, carbohydrates, proteins, lipids, and nucleic acids. Biofilms protect the fungi from environmental stresses, enhance cell cooperation, facilitate nutrient absorption, and reduce antifungal efficacy [36,37]. In vitro and ex vivo approaches have demonstrated that biofilm formation is associated with increased expression of genes related to pathogenesis and resistance, as well as reduced susceptibility to antifungals when compared to conidia [38,39]. In addition, Burkhart [40] proposed that dermatophytomas, dense and circumscribed dermatophytic white masses within the nail, are often associated with refractory onychomycosis. Although the formation of dermatophytic biofilms has been demonstrated, in vivo visualization has not yet been achieved, which hinders the understanding of the clinical impact of these structures in dermatophyte infections.

Considering all these factors, from species diversity and ecological niches to pathogenesis, it is clear that studying dermatophytosis is essential for understanding the factors that influence its high prevalence and wide range of clinical manifestations [8].

### 2.2. Clinical Manifestations of Dermatophytosis

Dermatophytes can infect several anatomical sites, primarily affecting the skin, hair, and nails. Clinical manifestations vary depending on the fungal species involved, the anatomical site affected, and the host’s immune response [4]. These infections are clinically classified according to the affected area and are designated as *tinea* followed by the infected site, such as *tinea capitis* (scalp), *tinea faciei* (face), *tinea barbae* (beard), *tinea corporis* (body), *tinea manuum* (hands), *tinea cruris* (groin), *tinea pedis* (feet), and *tinea unguium* (nails) [3].

*Tinea capitis* is a dermatophyte infection of the scalp, clinically characterized by localized alopecia, scaling, and brittle hair, which may progress to severe inflammatory forms such as kerion Celsi [41]. In these cases, an intense immune response can lead to painful, suppurative plaques. Without appropriate and targeted treatment, this process may damage the host’s hair follicles, replacing them with scar tissue where hair can no longer grow, ultimately leading to permanent scarring alopecia [42,43]. *Tinea faciei* affects the face, presenting with erythema and scaling, which is occasionally accompanied by papules and pustules [44]. In some instances, the infection extends to the beard area, affecting hair, skin, and hair follicles, manifesting as *tinea barbae*. This condition is clinically identified by non-inflammatory superficial plaques and, less frequently, by deep, inflammatory kerion Celsi-like plaques [45,46].

*Tinea corporis* is one of the most common clinical forms, typically affecting hairless areas and presenting as erythematous, scaly, ring-shaped lesions with raised, active borders, often accompanied by itching and central clearing [47]. *Tinea manuum* primarily affects the skin on the palms and interdigital finger of the hands, manifesting as diffuse scaling, hyperkeratosis, itchy vesicles, or reddish plaques with slightly raised borders [48].

*Tinea cruris* involves the inguinal region and skin folds, causing erythematous, scaly lesions, often associated with intense itching and discomfort. The infection often spreads to adjacent regions such as the perineum, buttocks, and inner thighs, particularly in immunocompromised individuals [49]. *Tinea pedis* typically affects the interdigital spaces between the toes of the feet and may present with scaling, fissures, maceration, and itching. Hyperkeratotic and vesicular forms may also occur [50]. *Tinea unguium* (onychomycosis) is one of the most persistent and recurrent forms, primarily affecting toenails. It is characterized by nail thickening, discoloration, brittleness, and onycholysis [51]. Its chronic course complicates treatment and leads to frequent relapses, especially in elderly, diabetic, and immunocompromised patients [4].

Although dermatophytosis is a cutaneous infectious disease, it can significantly impair patients’ quality of life, causing physical discomfort and aesthetic changes [5,52]. Delayed diagnosis can exacerbate these effects and have socioeconomic repercussions, posing an increased risk to immunocompromised patients, who are more susceptible to invasive forms of the disease [3,19,53]. To further complicate diagnosis, atypical forms of dermatophytosis, and uncommon species have also been reported [54,55].

Dermatophytosis has a broad clinical spectrum and is of significant epidemiological importance. The diversity of clinical manifestations reflects the complex pathogenesis of these infections, and knowledge of their various presentations directly contributes to early diagnosis, appropriate therapeutic selection, and effective management of dermatophytosis [8,56,57].

### 2.3. Antifungal Therapy for Dermatophytosis

The conventional treatment for dermatophytosis involves the use of topical or systemic antifungal agents, selected according to the lesion extent, the anatomical site affected, the etiological agent, and the patients’ previous therapeutic response. The main antifungal classes used to treat dermatophytosis include azoles, allylamines, amorolfine, ciclopirox olamine, and griseofulvin, either as monotherapy or in combination [8,58]. Combination therapy for dermatophytosis frequently involves a systemic antifungal agent administered orally together with a topical medication for several weeks, achieving effective clinical outcomes and representing a promising approach to accelerate both clinical and microbiological cure [59].

Topical and systemic azoles, such as itraconazole, fluconazole, clotrimazole, miconazole, and ketoconazole act by inhibiting lanosterol 14-α-demethylase, thereby impairing ergosterol biosynthesis and compromising fungal membrane integrity [60,61]. Among systemic azoles, itraconazole is particularly relevant for extensive or refractory dermatophytosis, especially in cases of terbinafine failure or suspected infection with resistant dermatophytes such as *T. indotineae* [8,62]. Allylamines, particularly terbinafine, inhibit squalene epoxidase, reducing ergosterol synthesis and promoting intracellular squalene accumulation, disrupting fungal growth and survival [63,64]. Amorolfine and ciclopirox olamine are available as nail lacquers. Amorolfine interferes with ergosterol biosynthesis by inhibiting both sterol-14-Δ reductase and sterol-Δ7-Δ8 isomerase, while ciclopirox olamine targets multiple metabolic processes, including mitochondrial function and metal-dependent enzymes, thereby increasing membrane permeability [65]. Although griseofulvin is currently less frequently used, it remains relevant mainly for treating *tinea capitis* infections. It acts by interference with microtubule function and fungal cell division [66,67].

Although these agents have been widely used for managing dermatophytosis, their use presents several limitations, including toxicity, limited availability (especially in low-income countries), high cost, poor adherence to long-term regimens, recurrence of infections, and antifungal resistance [68,69]. The emergence of resistant isolates of dermatophytes has gained increasing clinical importance. Studies have demonstrated the high susceptibility of *T. rubrum*, *T. mentagrophytes*, and *T. interdigitale* isolates to terbinafine, although elevated minimum inhibitory concentration (MIC) values have been reported for some isolates against itraconazole, miconazole, ciclopirox, and griseofulvin [70,71,72]. For *T. indotineae*, terbinafine resistance is a well-documented phenomenon, supported by robust studies involving large sample sizes, with resistance rates among clinical isolates ranging from 39% to 100%. In some studies, resistance to azoles has also been observed in terbinafine-resistant isolates [73,74,75,76,77]. For *M. canis*, resistance rates are generally low, although elevated MIC values have been reported for griseofulvin. In contrast, terbinafine and itraconazole have shown greater efficacy, representing suitable therapeutic options for managing *M. canis* infections [78,79].

Overall, resistance mechanisms involve the overexpression of efflux pumps, upregulation of stress-response signaling pathways, mutations in antifungal targets, and increased enzymatic activity of detoxification systems [80,81]. For example, mutations in the *SQLE* gene, which encodes the squalene epoxidase have been associated with increased MIC values and therapeutic failure in infections caused by *T. rubrum*, *T. mentagrophytes*, and *T. indotineae* [46,64,82]. Additionally, alterations in the ergosterol biosynthesis pathway and increased activity of efflux transporters may contribute to reduced susceptibility to azoles [62,63]. Moreover, overexpression of salicylate 1-monooxygenase leads to in vitro resistance to terbinafine, probably by drug degradation [83]. Therefore, accurate etiological identification, rational antifungal use, and, when possible, susceptibility testing are essential to guide therapeutic management, particularly in extensive or refractory cases, and limit the selection of resistant isolates [8,64].

Gaining insights into the complex dynamics of dermatophyte interaction with the host and the determinants of infection provides more effective prevention and treatment strategies, helping to mitigate the social, economic, and clinical burden of this widespread condition [8,57]. Furthermore, this knowledge can contribute to the development of improved diagnostic methods and optimization of therapeutic approaches, thereby reducing the risk of dissemination.

## 3. Epidemiology of Dermatophytosis

Dermatophytosis is a highly prevalent infection worldwide, mainly caused by *Trichophyton*, *Microsporum*, *Nannizzia*, and *Epidermophyton* species. The relative distribution of these pathogens is influenced by multiple host- and pathogen-related factors. Host-related factors such as sanitation conditions, socioeconomic status, immunocompetence, comorbidities, age, and gender have been reported as important determinants of transmission and disease progression [84,85,86]. Regarding the pathogens, ecological classification and genotype have been associated with infection progression, clinical manifestations, and in particular, recurrence [74,87].

**Figure 1:**
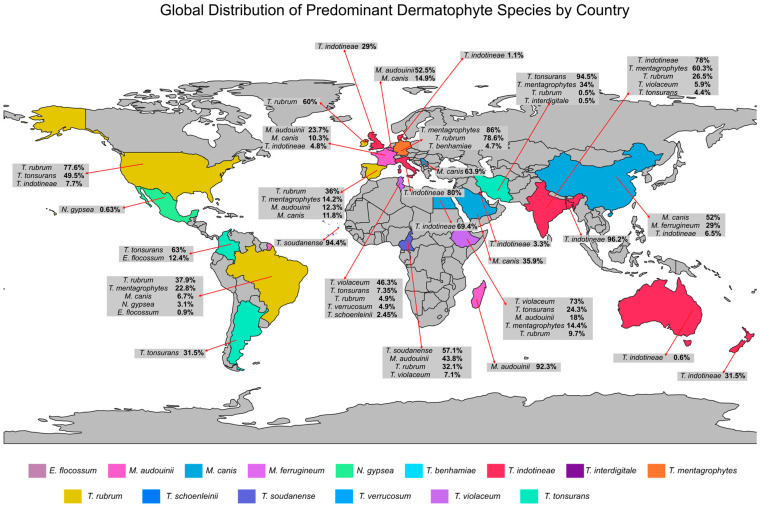
Global distribution of dermatophytes. Prevalence (%) of dermatophyte species in each country, according to the studies included in this review. Countries are color-coded based on the most frequently identified species, as described in the figure. The map was generated using QGIS software (version 4.0.2; QGIS Development Team).

Although several genera can cause dermatophytosis, *Trichophyton* spp. are reported as the primary causative agents in diverse geographic regions and study designs. Infections caused by non-*Trichophyton* species, including *Nannizzia*, *Epidermophyton*, and especially *Microsporum*, are less prevalent but have been associated with specific populations and transmission settings [88,89,90]. Figure 1 summarizes the prevalence of dermatophytes in each country, according to the studies included in this review.

### 3.1. Infections Caused by Trichophyton spp.

*Trichophyton* spp. have been consistently reported as the predominant etiological agents of dermatophytosis worldwide. The main findings of the studies discussed in this section are summarized in Table 1.

Studies conducted in Europe have demonstrated a predominance of *T. rubrum*, particularly in nail and foot infections. Aragón-Sánchez et al. [91] investigated the prevalence of dermatophytes in diabetic patients with onychomycosis and *tinea pedis* in Spain. Onychomycosis was confirmed in 40.6% of patients, and *T. rubrum* was identified as the most frequent etiological agent (36%), followed by *T. mentagrophytes* (14.2%). A similar pattern was observed by Powell et al. [48] in Ireland, where *T. rubrum* accounted for 60% of isolates from onychomycosis and *tinea pedis*. It also exhibited the highest prevalence in *tinea cruris* (83%) and *tinea manuum* (68%) cases, confirming its dominance across multiple anatomical sites.

Similar findings were reported by Kromer et al. [86], who found that *T. rubrum* comprised 78.6% of all dermatophyte infections in Germany. In contrast, the predominance of *T. rubrum* in nail and foot infections does not extend to scalp involvement. Across several European cohorts, *T. rubrum* plays a minor role in *tinea capitis*, whereas other *Trichophyton* species emerge as epidemiologically prominent. This difference may be partially explained by the distinct keratin composition of skin, nails, and hair, which influences dermatophyte tissue tropism and ability to colonize [92,93]. Indeed, *T. rubrum* is more frequently isolated from glabrous skin and nails, whereas other species, including *T. mentagrophytes*, *T. violaceum* and *Trichophyton soudanense*, are more commonly associated with scalp infections [86,94].

Importantly, several recent studies have revealed a significant prevalence of infections attributed to the *T. mentagrophytes/T. interdigitale* complex. In Germany, an analysis of 43 patients, most of whom suffered from highly inflammatory, painful, and persistent pubic and genital infections, was conducted to identify the causative agents. Sequencing results revealed *T. mentagrophytes* genotype VII to be the fungal agent in 86.0% of confirmed cases, followed by *T. benhamiae* (4.7%). Of the confirmed cases, 58.1% exhibited genital lesions and 18.6% presented with infections in multiple body areas [95]. Infections caused by species belonging to the *T. mentagrophytes/T. interdigitale* complex are difficult to identify and often require molecular analysis. However, an investigation conducted by Klinger et al. [84] in Switzerland demonstrated two distinct clinical profiles: *T. mentagrophytes* was more frequently associated with inflammatory, non-foot lesions in younger patients, whereas *T. interdigitale* was primarily linked to non-inflammatory foot and nail infections.

Several studies have reported cases of dermatophytosis caused by *Trichophyton* spp. in Asia. Kaul et al. [47] investigated the prevalence of dermatophytosis at a tertiary care hospital in India. They evaluated 300 cases and observed a high prevalence of *tinea corporis* (51.98%) and *tinea cruris* (31.44%). The predominant infections were caused by *T. mentagrophytes* (60.3%), followed by *T. rubrum* (26.5%), *T. violaceum* (5.9%), and *T. tonsurans* (4.4%). Similarly, *T. mentagrophytes* (34.0%) was the predominant agent in an Iranian national survey of patients with *tinea capitis* [85]. Notably, this study also reported reduced terbinafine susceptibility of two *T. mentagrophytes* isolates, which was associated with mutations in the *SQLE* gene.

Marked geographic and clinical heterogeneity is also evident in studies focusing on specific populations and transmission settings. For example, in contact sports, Kermani et al. [96] documented a predominance of *T. tonsurans* among Iranian wrestlers, accounting for 94.5% of all culture-positive isolates, while *T. rubrum* and *T. interdigitale* were rarely detected (≤1%). Firooz et al. [97] also reported two cases of chronic inguinal dermatophytosis caused by *T. tonsurans* in Iran. Notably, the isolates exhibited resistance to both terbinafine and fluconazole, representing a rare multidrug-resistant profile for *T. tonsurans*. This finding highlights the fact that antifungal resistance, although uncommon, can substantially complicate the clinical management of dermatophytosis. By contrast, an analysis of 313 patients with dermatophytosis in Kazakhstan revealed that 26.3% of infections were caused by *Trichophyton* spp., including *T. mentagrophytes*, *T. tonsurans*, and *T. verrucosum* [98].

A high incidence of *tinea capitis* has been reported in Africa, particularly in children. Bitew et al. [99] analyzed 301 clinical samples in Ethiopia and identified *T. violaceum* as the leading causative agent of *tinea capitis*, accounting for 73.0% of isolates, followed by *T. mentagrophytes* (9.5%) and *T. tonsurans* (8.5%). As expected, 77.0% of cases occurred in children aged 1–14 years. Diawara et al. [100] also investigated the prevalence of *tinea capitis* among children in 10 schools in the Republic of Guinea. A higher prevalence was observed in males (71.3%) and in children younger than 10 years (66.1%). Although identification was not performed at the species level, 91% of *tinea capitis* cases were associated with *Trichophyton* spp. Risk factor analysis revealed associations with male sex, home hairdressing, head shaving, family history, and proximity to household waste dumps. In addition, the use of antifungals, dermocorticoids, and antibacterials was also significantly associated with the occurrence of dermatophytosis.

Another study [101] conducted in Cameroon revealed a high prevalence of *T. soudanense* (57.1%) in *tinea capitis* among primary school children, while *T. rubrum* and *T. violaceum* were also documented at 32.1% and 7.1%, respectively. Interestingly, the authors reported that male sex, hairdressing at home, and regular head shaving were associated with an increased risk of *tinea capitis*. Similarly, Correia et al. [102] reported a high prevalence of infections caused by *T. soudanense* (94.4%) among 249 students in three rural schools in Cape Verde. These isolates were obtained mainly from cases of *tinea corporis* (33.33%) and onychomycosis (22.22%). Risk factor analysis revealed a higher prevalence of dermatophytosis among male students and individuals who reported taking up to four showers per week.

Although *tinea capitis* is typically associated with children, Chouaieb et al. [103] conducted a retrospective 14-year study in Tunisia analyzing adult cases. The most predominant agent was *T. violaceum* (46.3%), followed by *T. tonsurans* (7.35%), *T. verrucosum* (4.9%), *T. rubrum* (4.9%) and *Trichophyton schoenleinii* (2.45%). Based on 1580 confirmed cases, women were more frequently affected, particularly postmenopausal women. Notably, three cases, two caused by *T. tonsurans* and one by *T. verrucosum*, exhibited clinical resistance and yet they were successfully treated with terbinafine. *Tinea capitis* was also the predominant clinical manifestation in an analysis of hair, nail, and skin samples from patients with suspected dermatophytosis from Ethiopia [104]. It accounted for 53.4% of cases, followed by *tinea corporis* (30.5%) and onychomycosis (16%). *T. tonsurans* (40.2%), *T. mentagrophytes* (19.4%), and *T. rubrum* (9.7%) were the most common dermatophytes isolated, with *T. tonsurans* being the leading pathogen in *tinea capitis*, whereas *T. mentagrophytes* was most frequently associated with *tinea corporis*.

Infections caused by *Trichophyton* spp. are also prevalent in the Americas. Zarzeka et al. [105] investigated the causative agents of 17,589 cases of *tinea corporis* and *tinea cruris* in the United States. The authors observed a high prevalence of infections caused by *T. rubrum* and *T. tonsurans*, with *T. rubrum* being more frequently isolated from *tinea cruris* (77.6%), while *T. tonsurans* was the predominant agent in *tinea corporis* (49.5%).

*Tinea capitis* have also been documented in South America, particularly among children. Russo et al. [106] reported that *tinea capitis* infections had been documented in Argentina, particularly among children and adolescents, with *T. tonsurans* showing a significant prevalence (31.5%). All cases were associated with barbershop attendance, suggesting direct transmission of pathogens during haircuts. Similarly, González et al. [107] described an outbreak of *tinea capitis* in 32 children from a rural school in Colombia, where *T. tonsurans* was the predominant agent (63.0%). The main predisposing factor was sharing razor (87.5% of patients), although other habits, including sharing towels, combs, caps, beds, and having close contact with cats and dogs, were also common among affected individuals.

Two studies have investigated the prevalence of dermatophytosis in Brazil. Brito et al. [108] analyzed 2724 biological samples collected in Brazil. *T. rubrum* was the most frequently identified dermatophyte (68.6%), with a higher prevalence among patients aged 18–19 years, followed by *T. mentagrophytes* (21.4%). Interestingly, 86 individuals were found to have co-infections involving dermatophytes and other fungi were reported in, including combinations of *T. rubrum* with *Candida* spp., *Fusarium* spp., and *Neoscytalidium dimidiatum*, as well as *T. mentagrophytes* with *Candida* spp. or *Fusarium* spp., highlighting the ability of dermatophytes to coexist with other molds and yeasts. In another study, Correia et al. [109] investigated the epidemiology of dermatomycoses in children in Brazil. Among dermatophytes, species from the *T. mentagrophytes* represented 24.39% of isolates, while *T. rubrum* accounted for 7.28%. *T. mentagrophytes* was isolated from the interdigitoplantar skin, scalp, and toenails of patients aged 6 months to 12 years, with higher prevalence in children under 5 years. In contrast, *T. rubrum* was obtained from toenails and interdigitoplantar samples of patients aged 6–12 years.

Although *T. rubrum* remains the dominant species in onychomycosis and *tinea pedis* across multiple countries [48,91], the prevalence of other species, including *T. mentagrophytes*, *T. tonsurans*, and *T. violaceum*, has increased in recent years [86,94]. Moreover, this epidemiology landscape is influenced by several factors, including climate, age distribution, geography, comorbidities (e.g., diabetes), and outbreak-prone environments [48,85,86,110,111]. Importantly, several studies have reported the impact of climate change, natural events, and increased international travel on the global dissemination of fungi, including dermatophyte species [112,113,114]. These observations highlight the importance of identifying the causative species, both for accurate epidemiological surveillance and for optimized clinical management.

**Table 1:** Country, period of analysis, clinical presentations observed, and etiologic agents of infections caused by *Trichophyton* spp. included in the study.

Country	Period	Clinical Presentation	Etiological Agent	Reference
Spain	2016–2017	Onychomycosis and *tinea pedis*	*T. rubrum* and *T. mentagrophytes*	[91]
Switzerland	2001–2018	*Tinea* in multiple sites	*T. rubrum*, *T. interdigitale*, *T. violaceum*, *T. soudanense*, *T. tonsurans*, *T. mentagrophytes*, *T. benhamiae* and *T. verrucosum*	[94]
Switzerland	2009–2019	*Tinea* in multiple sites	*T. interdigitale* and *T. mentagrophytes*	[84]
Germany	2014–2016	*Tinea* in multiple sites	*T. rubrum*, *T. interdigitale*, *T. benhamiae*, *T. mentagrophytes* and *T. tonsurans*	[86]
Germany	2016–2017	*Tinea* in multiple sites	*T. equinum*, *T. interdigitale*, *T. tonsurans* and *T. mentagrophytes*	[95]
Ireland	2001–2020	*Tinea* in multiple sites	*T. rubrum*, *T. interdigitale*, *T. schoenleinii*, *T. soudanense*, *T. tonsurans*, *T. violaceum*, *T. mentagrophytes* and *T. verrucosum*	[48]
India	2022–2023	*Tinea* in multiple sites	*T. mentagrophytes*, *T. rubrum*, *T. violaceum* and *T. tonsurans*	[47]
Iran	2018–2019	*Tinea corporis*	*T. tonsurans*, *T. rubrum* and *T. interdigitale*	[96]
Iran	Not reported	*Tinea cruris*	*T. tonsurans*	[97]
Iran	2020–2021	*Tinea capitis*	*Trichophyton mentagrophytes*, *T. tonsurans*, *T. violaceum*, *T. benhamiae* and *T. schoenleinii*	[85]
Kazakhstan	2023–2023	*Tinea capitis*, *tinea corporis*, and *tinea cruris*	*T. mentagrophytes*, *T. tonsurans* and *T. verrucosum*	[98]
Ethiopia	2018–2019	*Tinea capitis*	*T. violaceum*, *T. mentagrophytes*, *T. tonsurans*, *T. verrucosum*, *T. schoenleinii*, and *T. soudanense*	[111]
Republic of Guinea	2021–2023	*Tinea capitis* and onychomycosis	*Trichophyton* spp.	[100]
Cameroon	2021	*Tinea capitis*	*T. soudanense*, *T. rubrum* and *T. violaceum*	[101]
Cape Verde	2020	*Tinea* in multiple sites	*T. soudanense* and *T. rubrum*	[102]
Tunisia	2009–2022	*Tinea capitis*	*T. violaceum*, *T. tonsurans*, *T. verrucosum*, *T. rubrum* and *T. schoenleinii*	[103]
Ethiopia	2019	*Tinea capitis*, *tinea corporis*, and onychomycosis	*T. tonsurans*, *T. mentagrophytes*, *T. rubrum*, *T. verrucosum*, *T. soudanense*, *T. violaceum* and *T. schoeninii*	[104]
United States	2019–2023	*Tinea corporis* and *tinea cruris*	*T. tonsurans* and *T. rubrum*	[105]
Argentina	2021–2023	*Tinea capitis*	*T. tonsurans*	[106]
Colombia	Not reported	*Tinea capitis*	*T. tonsurans*	[107]
Brazil	2015–2020	*Tinea* in multiple sites	*T. mentagrophytes* and *T. rubrum*	[109]
Brazil	2014–2020	*Tinea corporis*, onychomycosis, and *tinea pedis*	*T. rubrum*, *T. tonsurans* and *T. mentagrophytes*	[108]

### 3.2. Infections Caused by Trichophyton indotineae: An Emergent Dermatophyte

Although other *Trichophyton* species have historically been reported as the predominant agents of dermatophytosis, *T. indotineae* has emerged as a cause of infections associated with therapeutic failure. The first reports of *T. indotineae* originated from studies in South Asia, where cohorts of patients with extensive and recalcitrant dermatophytosis showed a high prevalence of infections [115]. Initially, the agent was described as *Trichophyton mentagrophytes* ITS genotype VIII, a strain within the *T. mentagrophytes*/*T. interdigitale* complex that was difficult to distinguish using conventional phenotypic methods. Subsequent molecular and genomic studies confirmed that this genotype represented a distinct phylogenetic entity, strongly associated with recalcitrant dermatophytosis and high rates of antifungal resistance. Ultimately, these findings led to its reclassification as a separate species, *T. indotineae*, thereby clarifying its epidemiological and clinical relevance [73,116,117].

Currently, epidemiological data from South Asia continues to indicate *T. indotineae* as the dominant agent in certain populations. In Bangladesh, Bhuiyan et al. [73] evaluated 99 patients with chronic, recalcitrant *tinea corporis* and found that 96.2% of the infections were caused by *T. indotineae* (96.2%). In this study, 64% of isolates were resistant to terbinafine, 28% to itraconazole, and 14% exhibited resistance to both agents. An analysis of patients with extensive dermatophytosis in Sri Lanka also revealed a high prevalence (50%) of *T. indotineae* infections [74]. Importantly, 80% of isolates exhibited resistance to terbinafine, 30% to clotrimazole, and all patients presented at least one relapse following clinical cure, reinforcing the recurrence pattern commonly associated with this species.

In another study, Ebert et al. [46] analyzed skin samples collected from 402 patients from eight locations across India. *T. indotineae* was identified in 314 samples (78%), with a high rate of terbinafine resistance (71%). Several retrospective and genomic studies suggest that South Asia, particularly the Indian subcontinent, is the primary reservoir of *T. indotineae*, with isolates from India and neighboring countries accounting for the largest proportion of reported cases worldwide, so far [7,116,117].

Beyond this epicenter, East Asia exhibits a distinct epidemiological profile, mainly characterized by exogenous cases with no evidence of sustained local transmission. In China, Jia et al. [117] examined 31 isolates from the *T. mentagrophytes*/*T. interdigitale* complex and identified *T. indotineae* in two cases (6.5%), both of which involved Indian nationals residing in China. Although one isolate exhibited terbinafine resistance, no onward transmission was observed during the study period. In the Middle East, a four-year retrospective study conducted in Kuwait identified *T. indotineae* in 3.3% of all dermatophyte isolates, mostly isolated from *tinea cruris* and *tinea corporis* cases, from patients aged 20–29 years and over 60. Although the frequency was low, its isolation in elderly patients, commonly associated with immunosenescence, raises concern due to the potential for multiple and extensive lesions [118].

In Europe, recent findings point to a gradual shift from sporadic imported cases towards established autochthonous transmission. In France, Moreno-Sabater et al. [119] evaluated 580 *Trichophyton* spp. isolates, of which 4.8% were *T. indotineae*, including one terbinafine-resistant isolate. Moreover, this species was responsible for 62.5% of *tinea corporis* cases, with a lower incidence (12.5%) in *tinea cruris*. Genomic analysis of these strains further supported the presence of European genotypes of terbinafine-resistant *Trichophyton* species, suggesting endogenous transmission in France. This local transmission also indicates that the pathogen can become endemic in certain areas, potentially leading to outbreaks. Such events are particularly concerning in infections caused by resistant strains or in immunosuppressed patients, as they may overwhelm healthcare systems and carry direct implications for public health [120,121].

An increasing prevalence of dermatophytosis caused by *T. indotineae* have also been reported in other European countries, including the United Kingdom, Denmark, Sweden, and Italy. The United Kingdom represents one of the most pronounced epidemiological scenarios on *T. indotineae* prevalence outside Asia. A national surveillance study conducted by Abdolrasouli et al. [122] in the UK documented a significant increase in the proportion of *T. indotineae* among dermatophytes, rising from 2% in 2018 to 38% in 2024. Clinically, extensive lesions involving more than five anatomical sites were observed in 54.2% of patients. Notably, 73.9% of patients had no history of travel, suggesting endogenous transmission. Among the tested isolates, 74.2% exhibited elevated in vitro resistance to terbinafine, raising concerns about resistance in this setting. Additionally, Czerniewska et al. [123] identified 363 unique cases and showed that *T. indotineae* has accounted for over 30% of all dermatophytes reported nationally, with evidence of household clusters and wide geographic dispersion, confirming sustained transmission. While these data largely derive from reference laboratories and may therefore over-represent refractory or previously treated cases, the consistence of the data over time and across the country supports a genuine expansion of the fungus within the country [75,122,123].

In Denmark, Astvad et al. [75] investigated 63 isolates and observed that *T. indotineae* accounted for 11.1% of *Trichophyton* isolates, representing a 250% increase in prevalence compared with the previous year. Furthermore, all *T. indotineae* isolates were resistant to terbinafine, suggesting an increasing prevalence of terbinafine-resistant *T. indotineae* isolates compared to previous studies [64]. In Italy, an investigation of five patients with *tinea cruris* and *tinea corporis* who failed to respond clinically to terbinafine treatment revealed that 80% of infections were caused by *T. indotineae* strains. Although terbinafine resistance was identified, all patients responded favorably to itraconazole therapy, indicating the recent introduction of the pathogen with tangible clinical consequences [77].

On the African continent, there is a single report of infection caused by *T. indotineae* [124]. The case described an extensive and difficult to treat dermatophytosis in a South African woman. Following diagnosis and pathogen identification based on sequencing of the *ITS* and the large subunit (*LSU*) gene regions, she was treated with oral griseofulvin, followed by oral terbinafine, with no clinical response. Subsequently, therapy was shifted to oral itraconazole combined with topical clotrimazole, which resulted in positive outcomes.

The limited number or absence of reports of dermatophytosis caused by *T. indotineae* across the African continent—despite the geographical proximity to epicenters of *T. indotineae* infections in Europe and Asia—is a matter of concern. This scarcity of data may reflect either the absence of the pathogen, its recent introduction or underdiagnosis of cases. To address this issue, Badiane et al. [125] conducted a survey across African countries to evaluate diagnostic capacity for cutaneous fungal diseases. Classical diagnostic methods were assessed in 47 countries. Notably, at least 15% of countries did not offer skin biopsy as an analytic method. Direct microscopy was not performed in 21%, fungal cultures were unavailable in at least 20% of countries, and histopathological examination was absent in 20%. Furthermore, no information was available regarding the status of molecular diagnostic methods for fungal diseases in these countries. The authors identified cost as a major limiting factor for the implementation of these diagnostic techniques.

This situation has been corroborated by studies investigating the prevalence of dermatophytosis in African countries, mainly among schoolchildren. Although data on incidence, anatomical sites affected, and risk factors have been reported, there is a lack of information on the specific pathogens responsible [126,127,128]. Importantly, due to the morphological similarity of *T. indotineae* with species from the *T. mentagrophytes/T. interdigitale* complex, conventional phenotypic methods are inadequate for its identification, necessitating molecular analysis [114]. Collectively, these findings may partially explain the underreporting of dermatophytosis caused by *T. indotineae* in African countries.

Few studies have reported the prevalence of *T. indotineae* in Oceania. A retrospective study of 961 dermatophyte isolates conducted in New Zealand revealed a significant prevalence of *T. indotineae* (9%). Among these isolates, 39% exhibited resistance to terbinafine, while 8% demonstrated resistance to itraconazole [76]. While itraconazole resistance remains uncommon among *T. indotineae* isolates, reduced susceptibility to itraconazole has already been reported in Bangladesh, Canada, and the United Kingdom [73,122,129]. This indicates that resistance to more than one antifungal may further limit treatment options for refractory dermatophytosis. In another study, Chua et al. [130] analyzed 2340 dermatophyte specimens in Australia and identified *T. indotineae* in 0.6% of samples. The isolates were obtained mainly from *tinea corporis* (36.4%), *tinea manuum* (18.2%) and *tinea cruris* (18.2%).

In the Americas, the epidemiology of *T. indotineae* reflects a comparatively earlier phase of emergence, with clear signs of ongoing spread. In the United States, the first cases were reported by Caplan et al. [131], including individuals with no history of international travel, suggesting a local transmission. In a subsequent report, Caplan et al. [132] evaluated 11 patients diagnosed with dermatophytosis caused by *T. indotineae*. All patients presented with widespread lesions that were unresponsive to topical therapy, and treatment with terbinafine failed in seven patients. Although nine patients reported previous travel to Bangladesh, whole-genome sequencing of US isolates revealed a distinct cluster that was genetically separated from Indian isolates.

The significant presence of *T. indotineae* in North America was also corroborated by a larger laboratory-based analysis conducted by Cañete-Gibas et al. [133], who collected 271 dermatophytes isolates. *T. indotineae* accounted for 7.7% of all dermatophytes and 42.9% of terbinafine-resistant isolates. Another study in Canada evaluated 47 cases of dermatophytosis due to *T. indotineae*, 71.4% of which showed resistance to terbinafine. Phylogenomic analysis revealed that 30.9% of the isolates clustered with sequences from South Asia, and 26.2% with isolates from New York, USA [129]. Importantly, neither geographic nor temporal clustering based on country was observed, suggesting exogenous transmission.

In Latin America, published data remain limited but already suggest recent introduction of *T. indotineae* into major urban centers. In Brazil, Almeida Jr. et al. [134] reported a confirmed case resistant to terbinafine in São Paulo. Similarly, Messina et al. [135] reported the first case of *tinea corporis* caused by *T. indotineae* in Argentina. Since the strain exhibited in vitro resistance to terbinafine, itraconazole-based therapy was prescribed, resulting in favorable clinical outcomes. The scarcity of reports from Latin America may partly reflect gaps in systematic molecular surveillance rather than the true absence of the pathogen. Accurate identification of *T. indotineae* remains challenging in many routine diagnostic laboratories due to its close morphological similarity to other members of the *T. mentagrophytes/T. interdigitale* complex, often requiring molecular methods for reliable differentiation. Consequently, regions where molecular diagnostics are not routinely implemented may underestimate the true burden of this emerging dermatophyte [6,134,136].

Finally, multinational genomic investigations underscore the global and recent nature of *T. indotineae* dissemination. In a study by dos Santos et al. [116], 347 isolates from 14 countries were analyzed, revealing low genomic diversity and absence of strict geographic clustering, consistent with recent international spread and multiple independent introductions. Approximately 65% of isolates were resistant to terbinafine. Indeed, several studies have demonstrated that resistance to terbinafine in *T. indotineae* is associated with mutations and amino acid substitutions at positions F397L, L393F and L393S in the *SQLE* gene [73,75,116,129,131]. In some cases, this genotype, although less frequent, also confers cross-resistance to other antifungal agents, including clotrimazole and itraconazole [73,74]. Although the number of publications on *T. indotineae* has increased since its initial report, global data on the genomic surveillance of isolates—particularly those harboring mutations conferring antifungal resistance—remain scarce. Such surveillance is crucial for determining whether isolates are of endogenous or exogenous origin, as well as for establishing strategies to control and manage potential outbreaks [113,114,119].

Data regarding the documents included in this review, along with their main information, are summarized in Table 2. Collectively, these findings reveal a consistent epidemiological pattern whereby *T. indotineae* emerges in South Asia [115], spreads through human mobility [76,116,133], and progressively establishes itself in new geographic settings. These cases are frequently accompanied by terbinafine resistance and local adaptation, posing an emerging challenge for the surveillance and management of dermatophytosis worldwide.

### 3.3. Infections Caused by Non-Trichophyton Dermatophyte Species

Although Trichophyton spp. are considered as the primary etiological agents of dermatophytosis worldwide, infections caused by Microsporum spp. and, to a lesser extent by Nannizzia and Epidermophyton spp., have also been globally reported [108,137,138]. Among dermatophytosis caused by non-Trichophyton species, Microsporum spp. stand out as the most frequently identified etiological agents, often associated with *tinea capitis* in pediatric populations [139,140,141]. The studies included in this section, together with their main information, are described in Table 3.

Studies conducted in Asia have revealed a significant prevalence of infections caused by several *Microsporum* species, including *M. canis*, *M. audouinii*, and *M. ferrugineum*. Alshehri et al. [142] analyzed 10,021 dermatological samples collected in Saudi Arabia, showing that 3.97% of infections were caused by dermatophytes, with *Microsporum* spp. representing 50.5% of isolates. These infections were more prevalent in patients under 10 years, with *M. canis* being the most common agent (35.9%).

Similarly, Zheng et al. [141] investigated 171 patients diagnosed with *tinea capitis* in China, identifying *M. canis* (62%) as the predominant pathogen, with cases being more frequent in children aged 2–8 years (74.3%). Another study in China, analyzing 198 patients with *tinea capitis*, revealed a high prevalence in children (96%), particularly preschoolers aged 3–5 years (54%). In this cohort, *M. canis* was the most common dermatophyte (42%), followed by *M. ferrugineum* (29%) [143]. Albeit *M. canis* predominates in China, a recent study reported the first case of *tinea capitis* caused by *M. audouinii* in the country, involving three members of an African family who had been living in China for one year [140].

On the African continent, several studies have identified *M. audouinii* as the leading dermatophyte causing *tinea capitis*. A clinical-epidemiological survey conducted in Madagascar revealed a *tinea capitis* prevalence of 8.7%, with the most affected age group between 8 and 11 years, and *M. audouinii* identified in 92.3% of cases [139]. Another study performed in Cameroon examined 1070 students aged 5–13 years and found that 10.1% had *tinea capitis* indicative lesions. Among the 32 dermatophytes isolated, *M. audouinii* was the most frequent (43.8%) [144].

Araya et al. [104] performed a cross-sectional study of hair, nail, and skin samples in Ethiopia and identified *M. audouinii* as the etiological agent in 18% of cases. Importantly, *M. audouinii* exhibited a significantly higher prevalence in *tinea corporis* (23.8%) cases, followed by *tinea capitis* (17.3%). Another investigation of *tinea capitis* among children in the Republic of Guinea [100] revealed a prevalence of 9% of infections caused by *Microsporum* spp. Although *M. audouinii* has been predominantly isolated in Africa, its increasing reports in other continents are likely associated with population migration [89].

In contrast with other studies conducted in Africa, Farag et al. [145] reported *M. canis* as the predominant agent among schoolchildren in Egypt. Positive cultures were obtained in 88.6% of *tinea capitis* cases and 75% of *tinea corporis* cases, with *M. canis* identified in 69.4% of samples. As expected, a higher prevalence of infection was observed among boys, those living in low socio-economic conditions, and individuals with a family history of dermatophyte infections. Pet contact, as well as sharing towels and caps, were identified as significant risk factors for infection.

An epidemiological investigation conducted in Serbia revealed a 2.5% prevalence of fungal infections among the 1643 analyzed patients. Within the dermatophyte group, *M. canis* was the most prevalent species (63.9%) [146]. Pablo-Hernando et al. [147] investigated dermatophyte positivity in 4371 samples collected in Spain, reporting a detection rate of 16.7%. Among the non-*Trichophyton* species, *M. canis* (11.8%) was the most prevalent, followed by *M. audouinii* (3.8%). Statistically significant associations were observed between age and clinical presentation, with *tinea capitis* and *tinea corporis* being more frequent in individuals under 16 years of age.

Although *M. canis* has historically been the leading cause of *tinea capitis* in children, recent epidemiological studies in Europe have reported a shift in species distribution, with an increasing prevalence of *M. audouinii*, particularly in regions with significant migratory flows [148]. In France, an analysis of immigrant children revealed 1311 positive dermatophyte cultures, of which 28.2% were *M. audouinii* [149]. Another study in France investigated 4395 cases of *tinea capitis* over a six-year period reporting that 19.2% were caused by *M. audouinii*. In contrast, *M. canis* was less prevalent, accounting for 454 cases (10.3%). The highest incidence of *tinea capitis* was observed in children aged 0–10 years (85.6%) [150]. Similar findings were reported in an outbreak in Sweden, where 54 individuals (median age 6 years) tested positive for *M. audouinii*. Notably, terbinafine showed a high rate of treatment failure, whereas griseofulvin achieved successful clinical and mycological outcomes [151]. A study by Sacheli et al. [62] in Belgium evaluated 14,271 positive fungal cultures collected over a five-year period and identified 6169 cases of dermatophytosis. Among the etiological agents of *tinea capitis*, *M audouinii* was the most frequent (52.5%), followed by *M. canis* (14.9%).

In Denmark, a *tinea capitis* outbreak in a childcare facility showed that 10 out of 73 examined individuals were infected with *M. audouinii*. Although griseofulvin is considered the first-line treatment for *tinea capitis* caused by *Microsporum* spp., treatment failure was observed in 66% of cases in this study. In such cases, antifungal therapy was switched to alternative agents, including terbinafine, itraconazole, or fluconazole, leading to mycological cure [152]. Similarly, Campayo et al. [90] reported a terbinafine resistance rate of 17% among patients diagnosed with *tinea capitis* in Spain. Most patients (83.3%) were born in Africa and/or had direct contact with relatives residing there. In this study, *M. audouinii* was the second most prevalent dermatophyte overall and the most prevalent species among the non-*Trichophyton* dermatophytes (20.8%). Notably, three patients infected with *M. audouinii* showed no clinical improvement after treatment with terbinafine, which was subsequently replaced by griseofulvin.

As has been observed in other regions, non-*Trichophyton* dermatophytes have also been reported in the Americas, albeit at lower frequencies. Brito et al. [108] evaluated 7927 biological samples in Brazil and detected dermatophytes in 52.6% of the samples analyzed. Among the non-*Trichophyton* species identified, *M. canis* was the most frequent (6.7%), followed by *Nannizzia gypsea* (formerly *Microsporum gypseum*) (3.1%) and *Epidermophyton floccosum* (0.9%). Although *M. audouinii* had not been previously reported as a causative agent of *tinea capitis* in South America until 2017, Santino et al. [153] described a series of *tinea capitis* cases caused by this species in Brazil, with patients having a mean age of 6.1 years.

In addition to infections caused by *Microsporum* spp., cases of dermatophytosis due to *Nannizzia* and *Epidermophyton* species have also been described, although they remain rare. Orozco-Yee et al. [138] analyzed 24,449 cases of dermatophytosis in Mexico and observed that 0.63% were attributable to *N. gypsea*. This infection predominantly occurred in children aged 1–10 years (54.2%). Another study in Taiwan reported two cases of *tinea* caused by *Nannizzia polymorpha*. One patient, a 10-year-old boy, was initially treated with oral griseofulvin, which was later replaced by oral terbinafine due to suspected allergic reaction. After two weeks, terbinafine was discontinued because of persistent adverse effects. The second patient, a 68-year-old woman diagnosed with *tinea manuum*, was treated with oral terbinafine and topical ciclopirox cream, achieving mycological cure [154]. Soankasina et al. [155] also reported a case of a patient in Madagascar presenting with moderate inflammation of a discreetly squamous lesion located on the abdomen. A detailed medical history revealed a permanent habit of sleeping with cats. Sequencing of the *ITS* region confirmed *N. gypsea* as the pathogen. Treatment with topical miconazole cream resulted in complete recovery.

Similarly, infections caused by species of the genus *Epidermophyton* have also been sporadically reported in recent years. Epidemiological studies indicate that *E. floccosum* typically accounts for a smaller proportion of dermatophyte isolates than species of the genus *Trichophyton* [156]. However, in a 23-year retrospective study conducted in Colombia, *E. floccosum* accounted for 12.4% of dermatophyte isolates, ranking it as the third most frequently identified species [137].

Despite its relatively low prevalence in most epidemiological surveys, sporadic clinical cases continue to be reported. For instance, Besrour et al. [88] described two cases of dermatophytosis caused by *E. floccosum* in patients treated in Tunisia. *E. floccosum*, a dermatophyte that primarily causes skin and nail infections in humans, has shown a declining trend in recent years. In this report, a 70-year-old diabetic patient with *tinea pedis* due to *E. floccosum* was treated with topical terbinafine, showing a favorable clinical outcome. Another 70-year-old patient diagnosed with *tinea unguium* was treated with oral terbinafine, also leading to mycological cure.

Considering non-*Trichophyton* dermatophyte species, *Microsporum* spp. are the most common cause of infections, often associated with *tinea capitis* in children [157]. The incidence of this disease decreases after puberty due to the protective and fungistatic properties of sebum [158]. In addition to the age-related factors, the prevalence of *tinea capitis* varies across geographical regions and is further influenced by factors such as overcrowded environments with poor hygiene and sanitation, humid and temperate climates, nutritional deficiencies, rural residence, frequent contact with animals, and other immunodeficiencies [159,160]. Although prevalence remains relatively low, the increasing number of infections caused by resistant species underscores the importance and urgency in establishing standardized protocols for managing dermatophytic infections, particularly those caused by less common species such as *M. audouinii*. This context also reinforces the importance of monitoring epidemiological data worldwide, with a focus on susceptibility testing and genomic analysis to track the circulation of these species between countries and, eventually, across continents.

**Table 3:** Country, period of analysis, clinical presentations observed, and etiologic agents of infections caused by non-*Trichophyton* dermatophyte species.

Country	Period	Clinical Presentation	Etiological Agent	Reference
Saudi Arabia	2000–2019	*Tinea corporis*, *tinea pedis*, *tinea unguium*, and *tinea capitis*	*Microsporum* spp.	[142]
China	2019–2022	*Tinea capitis*	*Microsporum canis*	[141]
China	2010–2021	*Tinea capitis*	*M. canis* and *Microsporum ferrugineum*	[143]
China	2025	*Tinea capitis*	*Microsporum audouinii*	[140]
Taiwan	2023	*Tinea capitis* and *tinea manuum*	*Nannizzia polymorpha*	[154]
Madagascar	2025	*Tinea capitis*	*M. audouinii*	[139]
Cameroon	2021	*Tinea capitis*	*M. audouinii*	[144]
Republic of Guinea	2021–2023	*Tinea capitis* and onychomycosis	*Microsporum* spp.	[100]
Ethiopia	2019	*Tinea capitis*, *tinea corporis*, and onychomycosis	*M. audouinii*	[104]
Egypt	2015–2016	*Tinea capitis*, *tinea pedis*, *tinea corporis* and onychomycosis	*M. canis*	[145]
Madagascar	2017	*Tinea corporis*	*N. gypsea*	[155]
Tunisia	2025	*Tinea pedis* and onychomycosis	*Epidemorphyton floccosum*	[88]
Serbia	2012–2017	*Tinea corporis* and *tinea capitis*	*M. canis*	[146]
Spain	2004–2019	*Tinea capitis*	*M. audouinii*	[90]
Spain	2020–2023	*Tinea corporis* and *tinea capitis*	*M. canis* and *M. audouinii*	[147]
Sweden	2019	*Tinea capitis*	*M. audouinii*	[151]
France	2014–2020	*Tinea capitis*	*M. audouinii* and *M. canis*	[150]
Belgium	2012–2016	*Tinea capitis*	*M. audouinii* and *M. canis*	[62]
Denmark	2024	*Tinea capitis*	*M. audouinii*	[152]
Brazil	2014–2020	*Tinea corporis*, onychomycosis, and *tinea pedis*	*M. canis*, *Nannizzia gypsea*, and *E. floccosum*	[108]
Brazil	2012–2019	*Tinea capitis*	*M. audouinii*	[153]
Mexico	2001–2023	*Tinea corporis* and *tinea capitis*	*N. gypsea*	[138]
Colombia	1994–2016	*Tinea pedis* and *tinea corporis*	*E. floccosum*	[137]

## 4. Conclusions

The global epidemiology of dermatophytes has been investigated in several studies conducted across different countries. Consistently with previous reports, *Trichophyton* spp. remain the predominant pathogens worldwide, with *T. rubrum*, *T. mentagrophytes*, and *T. interdigitale* being the most prevalent species. In contrast, *T. tonsurans* and *T. violaceum* have been reported as prominent agents in studies focusing on specific populations and transmission settings, particularly in cases of *tinea capitis*.

Multinational genomic investigations have highlighted the global dissemination of *T. indotineae*, confirmed by several endogenous cases reported in Asia and Europe, and exogenous reports in the Americas and Oceania. Notably, a high prevalence of in vitro resistance to terbinafine in several strains due to mutations in *SQLE* has been reported, with resistance also being confirmed in vivo. These studies corroborate the reports of infections associated with widespread and extensive lesions, therapeutic failure, unfavorable clinical outcomes, and recurrence. Moreover, sequencing and PCR have been demonstrated to be the gold standard methods for identifying *T. indotineae*, as classical approaches have proven ineffective for accurate identification of this species. In this context, the reduced number or absence of reports of *T. indotineae* infections in the African continent deserves attention, given both the geographical proximity to continents with a higher incidence of infections caused by this pathogen and the limitations of diagnostic methods identified across several African countries.

Among non-*Trichophyton* dermatophyte species, *Microsporum canis* and *M. audouinii* stand out as relevant pathogens, particularly in cases of *tinea capitis*. Despite their lower overall prevalence, these species have been described as predominant pathogens in some studies, surpassing *Trichophyton* spp. In this context, it is important to highlight the shift in the prevalence profile of pathogens associated with *tinea capitis* in Europe, where the prevalence of *M. canis* has decreased, while *M. audouinii* has become more common in regions experiencing significant migratory flows, with infections linked to treatment failure and notable rates of antifungal resistance.

Altogether, these findings reinforce the importance of global vigilance concerning dermatophyte dissemination, particularly for the surveillance of mutations and resistant phenotypes. This review also reveals a lack of recent data on dermatophyte prevalence in several countries, highlighting a gap between accurate fungal identification and global reporting, a matter of concern for epidemiological surveillance and the development of effective strategies for managing dermatophytosis worldwide. In this context, expanding efforts to establish standardized antifungal susceptibility testing for dermatophytes, training human resources to provide accurate diagnosis of these pathogens, and implementing molecular surveillance networks could help minimize these gaps and increase the reliability of global fungal epidemiological data.

## Figures and Tables

**Table 2:** Country, period of analysis, and clinical presentations of infections caused by *Trichophyton indotineae* included in the study.

Country	Period	Clinical Presentation	Reference
Bangladesh	Not informed	*Tinea corporis*	[73]
Sri Lanka	Not informed	*Tinea* in multiple sites	[74]
India	2017–2019	*Tinea corporis*, *tinea cruris*, *tinea faciei*, *tinea manuum*, *tinea pedis*, and *tinea barbae*	[46]
China	2017–2022	*Tinea corporis*, *tinea cruris*, and *tinea faciei*	[117]
Kuwait	2021–2024	*Tinea cruris* and *tinea corporis*	[118]
France	2021	Onychomycosis, *tinea pedis*, *tinea manuum*, *tinea cruris*, and *tinea corporis*	[119]
United Kingdom	2017–2024	*Tinea* in multiple sites	[122]
United Kingdom	2017–2025	*Tinea* in multiple sites	[123]
Denmark	2019–2020	Not informed	[75]
Italy	2019–2022	*Tinea corporis* and *tinea cruris*	[77]
South Africa	2021	*Tinea cruris*	[124]
New Zealand	2017–2024	*Tinea* in multiple sites	[76]
Australia	2024	*Tinea corporis*, *tinea cruris*, *tinea manuum*, *tinea pedis*, and *tinea capitis*	[130]
United States	2021–2023	*Tinea corporis* and *tinea cruris*	[132]
United States and Canada	2017–2022	Not informed	[133]
Canada	2014–2023	Not informed	[129]
Brazil	2024	*Tinea cruris*	[134]
Argentina	Not informed	*Tinea corporis*	[135]
Multiple countries	2016–2023	Not informed	[116]

## Data Availability

The original contributions presented in this study are included in the article. Further inquiries can be directed to the corresponding author(s).

## References

[1] Machová L., Kostovčík M., Švec K., Hubka V., Kolařík M., Wennrich A. (2025). Comparative gene expression analysis in closely related dermatophytes reveals secondary metabolism as a candidate driver of virulence. Microbiol. Spectr..

[2] Ciesielska A., Kawa A., Kanarek K., Soboń A., Szewczyk R. (2021). Metabolomic analysis of *Trichophyton rubrum* and *Microsporum canis* during keratin degradation. Sci. Rep..

[3] Moskaluk A.E., VandeWoude S. (2022). Current Topics in Dermatophyte Classification and Clinical Diagnosis. Pathogens.

[4] Deng R., Wang X., Li R. (2023). Dermatophyte infection: From fungal pathogenicity to host immune responses. Front. Immunol..

[5] Narang T., Bhattacharjee R., Singh S., Jha K., Kavita Mahajan R., Dogra S. (2019). Quality of life and psychological morbidity in patients with superficial cutaneous dermatophytosis. Mycoses.

[6] Uhrlaß S., Verma S.B., Gräser Y., Rezaei-Matehkolaei A., Hatami M., Schaller M., Nenoff P. (2022). *Trichophyton indotineae*—An Emerging Pathogen Causing Recalcitrant Dermatophytoses in India and Worldwide—A Multidimensional Perspective. J. Fungi.

[7] Gupta A.K., Susmita Nguyen H.C., Liddy A., Talukder M., Wang T., Magal L., Chowdhary A., Shemer A., Saunte D.M.L., Hay R. (2026). *Trichophyton indotineae*: Epidemiology, antifungal resistance and antifungal stewardship strategies. J. Eur. Acad. Dermatol. Venereol..

[8] Jartarkar S.R., Patil A., Goldust Y., Cockerell C.J., Schwartz R.A., Grabbe S., Goldust M. (2021). Pathogenesis, Immunology and Management of Dermatophytosis. J. Fungi.

[9] Dubljanin E., Zunic J., Vujcic I., Colovic Calovski I., Sipetic Grujicic S., Mijatovic S., Dzamic A. (2024). Host-Pathogen Interaction and Resistance Mechanisms in Dermatophytes. Pathogens.

[10] Rodriguez-Temporal D., Adrados D., Alastruey-Izquierdo A., Alkorta M., Candela A., Canut A., Castro C., Cilla C.G., Caballero J.D., Rodríguez-Sánchez B. (2025). Current Performance of MALDI–TOF Mass Spectrometry Databases for the Identification of Dermatophyte Species. J. Fungi.

[11] de Hoog G.S., Dukik K., Monod M., Packeu A., Stubbe D., Hendrickx M., Kupsch C., Stielow J.B., Freeke J., Göker M. (2017). Toward a Novel Multilocus Phylogenetic Taxonomy for the Dermatophytes. Mycopathologia.

[12] Baert F., Stubbe D., D’hooge E., Packeu A., Hendrickx M. (2019). Updating the Taxonomy of Dermatophytes of the BCCM/IHEM Collection According to the New Standard: A Phylogenetic Approach. Mycopathologia.

[13] Gupta A.K., Liddy A., Megal L., Kaplan B., Shemer A., Saunte D.M.L., Wang T. (2025). Sexually Transmitted Dermatophyte Infections—A Scoping Review. Mycoses.

[14] Peres N.T.d.A., Maranhão F.C.A., Rossi A., Martinez-Rossi N.M. (2010). Dermatophytes: Host-pathogen interaction and antifungal resistance. An. Bras. Dermatol..

[15] Sardana K., Gupta A., Mathachan S.R. (2021). Immunopathogenesis of Dermatophytoses and Factors Leading to Recalcitrant Infections. Indian Dermatol. Online J..

[16] Chiriac A., Diaconeasa A., Voicu C., Ivaniciuc M., Miulescu R., Chiriac A.E., Nenoff P., Wollina U. (2024). Kerion Celsi in infants and children—A narrative review 2010–2023. Mycoses.

[17] Baumbach C.M., Müller S., Reuschel M., Uhrlaß S., Nenoff P., Baums C.G., Schrödl W. (2021). Identification of Zoophilic Dermatophytes Using MALDI-TOF Mass Spectrometry. Front. Cell. Infect. Microbiol..

[18] Gnat S., Łagowski D., Nowakiewicz A. (2021). Genetic Predisposition and its Heredity in the Context of Increased Prevalence of Dermatophytoses. Mycopathologia.

[19] Dellière S., Jabet A., Abdolrasouli A. (2024). Current and emerging issues in dermatophyte infections. PLoS Pathog..

[20] Gnat S., Nowakiewicz A., Łagowski D., Zięba P. (2019). Host- and pathogen-dependent susceptibility and predisposition to dermatophytosis. J. Med. Microbiol..

[21] Kaplan E., Gonca S., Kandemir H., Döğen A., Hilmioğlu-Polat S., Ilkit M., Tanaka R., Yaguchi T., Uhrlaβ S., Nenoff P. (2019). Genes Encoding Proteolytic Enzymes Fungalysin and Subtilisin in Dermatophytes of Human and Animal Origin: A Comparative Study. Mycopathologia.

[22] Faway É., Poirier W., Yamada T., Ozawa K., Monod M., Mignon B., Poumay Y. (2025). *SUB6* Subtilisin is Involved During the Initial Adhesion of *Trichophyton benhamiae* and *T. mentagrophytes* onto Reconstructed Human Epidermis. JID Innov..

[23] Naeimipour F., Hashemi S.J., Rezaie S., Bayat M. (2021). Subtilisin Gene Activity in Dermatophytes: A study on the Presence of the Subtilisin Gene in *Trichophyton verrucosum* and *Microsporum gypseum* in Clinical and Nonclinical Samples in Tehran, Iran. Arch. Razi Inst..

[24] Łagowski D., Gnat S., Nowakiewicz A., Osińska M. (2021). Assessment of the subtilisin gene profile in *Trichophyton verrucosum* isolated from human and animal dermatophytoses in two-stage multiplex PCR. J. Appl. Microbiol..

[25] Méhul B., Gu Z., Jomard A., Laffet G., Feuilhade M., Monod M. (2016). *Sub6* (Tri r 2), an Onychomycosis Marker Revealed by Proteomics Analysis of *Trichophyton rubrum* Secreted Proteins in Patient Nail Samples. J. Investig. Dermatol..

[26] Lindenhahn J., Bartosch T., Baumbach C.M., Suchowski M., Kacza J., Schrödl W., Michler J.K. (2021). Detection of subtilisin 3 and 6 in skin biopsies of cattle with clinically manifested bovine ringworm. Med. Mycol..

[27] Gupta C., Das S., Gaurav V., Singh P.K., Rai G., Datt S., Tigga R.A., Pandhi D., Bhattacharya S.N., Ansari M.A. (2023). Review on host-pathogen interaction in dermatophyte infections. J. Med. Mycol..

[28] Grumbt M., Monod M., Yamada T., Hertweck C., Kunert J., Staib P. (2013). Keratin Degradation by Dermatophytes Relies on Cysteine Dioxygenase and a Sulfite Efflux Pump. J. Investig. Dermatol..

[29] Neves-da-Rocha J., Bitencourt T.A., de Oliveira V.M., Sanches P.R., Rossi A., Martinez-Rossi N.M. (2019). Alternative Splicing in Heat Shock Protein Transcripts as a Mechanism of Cell Adaptation in *Trichophyton Rubrum*. Cells.

[30] Jacob T.R., Peres N.T.A., Martins M.P., Lang E.A.S., Sanches P.R., Rossi A., Martinez-Rossi N.M. (2015). Heat Shock Protein 90 (Hsp90) as a Molecular Target for the Development of Novel Drugs Against the Dermatophyte *Trichophyton rubrum*. Front. Microbiol..

[31] Bitencourt T.A., Lang E.A.S., Sanches P.R., Peres N.T.A., Oliveira V.M., Fachin A.L., Rossi A., Martinez-Rossi N.M. (2020). HacA Governs Virulence Traits and Adaptive Stress Responses in *Trichophyton rubrum*. Front. Microbiol..

[32] Peres N.T.A., Lang E.A.S., Bitencourt T.A., Oliveira V.M., Fachin A.L., Rossi A., Martinez-Rossi N.M. (2022). The bZIP Ap1 transcription factor is a negative regulator of virulence attributes of the anthropophilic dermatophyte *Trichophyton rubrum*. Curr. Res. Microb. Sci..

[33] Bitencourt T.A., Neves-da-Rocha J., Martins M.P., Sanches P.R., Lang E.A.S., Bortolossi J.C., Rossi A., Martinez-Rossi N.M. (2021). StuA-Regulated Processes in the Dermatophyte *Trichophyton rubrum*: Transcription Profile, Cell-Cell Adhesion, and Immunomodulation. Front. Cell. Infect. Microbiol..

[34] Burstein V.L., Beccacece I., Guasconi L., Mena C.J., Cervi L., Chiapello L.S. (2020). Skin Immunity to Dermatophytes: From Experimental Infection Models to Human Disease. Front. Immunol..

[35] Martinez-Rossi N.M., Peres N.T.A., Rossi A. (2017). Pathogenesis of Dermatophytosis: Sensing the Host Tissue. Mycopathologia.

[36] Mendonça M.B., Cabral A.K.L.F., Arantes B.B.A., dos Santos R.C., Medina-Alarcón K.P., dos Santos K.S., Gualque M.W.L., Fernandes L.G., Belizario J.A., Fuzinaga T.Y.T. (2025). Biofilm development in three-dimensional models infected with *Trichophyton rubrum*. Microbiol. Spectr..

[37] Markantonatou A.M., Samaras K., Vyzantiadis T.A. (2023). Dermatophytic Biofilms: Characteristics, Significance and Treatment Approaches. J. Fungi.

[38] Yazdanpanah S., Sasanipoor F., Khodadadi H., Rezaei-Matehkolaei A., Jowkar F., Zomorodian K., Kharazi M., Mohammadi T., Nouripour-Sisakht S., Nasr R. (2023). Quantitative analysis of *in vitro* biofilm formation by clinical isolates of dermatophyte and antibiofilm activity of common antifungal drugs. Int. J. Dermatol..

[39] Arantes B.B.A., Cabral A.K.L.F., dos Santos K.S., Mendonça M.B., dos Santos R.C., Bugalho B.C.M., Fernandes L.S., Martinez L.R., Fusco-Almeida A.M., Mendes-Giannini M.J.S. (2025). Characterization of Biofilm Formation by the Dermatophyte *Nannizzia gypsea*. J. Fungi.

[40] Burkharta C.N., Burkhart C.G., Gupta A.K. (2002). Dermatophytoma: Recalcitrance to treatment because of existence of fungal biofilm. J. Am. Acad. Dermatol..

[41] Gupta A.K., Friedlander S.F., Simkovich A.J. (2022). *Tinea capitis:* An update. Pediatr. Dermatol..

[42] Zhan P., Liu W. (2017). The Changing Face of Dermatophytic Infections Worldwide. Mycopathologia.

[43] John A.M., Schwartz R.A., Janniger C.K. (2018). The kerion: An angry *Tinea capitis*. Int. J. Dermatol..

[44] Khiewplueang K., Leeyaphan C., Bunyaratavej S., Jirawattanadon P., Saengthong-aram P., Matthapan L., Prasong W., Panyawong C., Plengpanich A. (2024). *Tinea faciei* clinical characteristics, causative agents, treatments and outcomes; a retrospective study in Thailand. Mycoses.

[45] Kuruvella T., Saleh H.M., Pandey S. (2026). Tinea Barbae.

[46] Ebert A., Monod M., Salamin K., Burmester A., Uhrlaß S., Wiegand C., Hipler U., Krüger C., Koch D., Wittig F. (2020). Alarming India-wide phenomenon of antifungal resistance in dermatophytes: A multicentre study. Mycoses.

[47] Kaul N., Kumari N., Rawat S.D.S., Roy R., Nautiyal S. (2026). Clinicomycological and Dermoscopic Study of 300 Cases of Dermatophytosis in a Tertiary Care Hospital in Dehradun, India. Indian J. Dermatol..

[48] Powell J., Porter E., Field S., O’Connell N.H., Carty K., Dunne C.P. (2022). Epidemiology of dermatomycoses and onychomycoses in Ireland (2001–2020): A single-institution review. Mycoses.

[49] Sahoo A., Mahajan R. (2016). Management of *tinea corporis, tinea cruris,* and *tinea pedis*: A comprehensive review. Indian Dermatol. Online J..

[50] Leung A.K., Barankin B., Lam J.M., Leong K.F., Hon K.L. (2023). *Tinea pedis*: An updated review. Drugs Context.

[51] Lipner S.R., Scher R.K. (2019). Onychomycosis. J. Am. Acad. Dermatol..

[52] Kovitwanichkanont T., Chong A. (2019). Superficial fungal infections. Aust. J. Gen. Pract..

[53] Guilherme de Oliveira N., do Carmo de Camargo D., Bortolomai B.E., Ferreira Batista L.C., Foschiani Dias Baptista I.M. (2025). Epidemiology and geospatial distribution of dermatophyte fungi in the State of São Paulo, Brazil. Hygeia Rev. Bras. Geogr. Médica Saúde.

[54] Barak Levitt J.A., Kulish N., Hamed M., Ziv M., Barak E.C. (2026). Atypical Dermatophytosis in Patients Treated by JAK Inhibitors. Exp. Dermatol..

[55] Markwitz M., Łabędź N., Welc N., Kanabaj K., Bowszyc-Dmochowska M., Kubisiak-Rzepczyk H., Dańczak-Pazdrowska A. (2026). *Tinea Incognito* Imitating Pustular Psoriasis in a Psoriatic Patient Following Immunosuppressive Therapy: Case Report and Narrative Review. J. Clin. Med..

[56] Hayette M.P., Sacheli R. (2015). Dermatophytosis, Trends in Epidemiology and Diagnostic Approach. Curr. Fungal Infect. Rep..

[57] Mushtaq S., Faizi N., Amin S.S., Adil M., Mohtashim M. (2020). Impact on quality of life in patients with dermatophytosis. Australas. J. Dermatol..

[58] Gupta A.K., Cooper E.A. (2008). Update in antifungal therapy of dermatophytosis. Mycopathologia.

[59] Brescini L., Fioriti S., Morroni G., Barchiesi F. (2021). Antifungal Combinations in Dermatophytes. J. Fungi.

[60] Barac A., Stjepanovic M., Krajisnik S., Stevanovic G., Paglietti B., Milosevic B. (2024). Dermatophytes: Update on Clinical Epidemiology and Treatment. Mycopathologia.

[61] Monk B.C., Sagatova A.A., Hosseini P., Ruma Y.N., Wilson R.K., Keniya M.V. (2020). Fungal Lanosterol 14α-demethylase: A target for next-generation antifungal design. Biochim. Biophys. Acta (BBA)-Proteins Proteom..

[62] Sacheli R., Cuypers L., Seidel L., Darfouf R., Adjetey C., Lagrou K., Hayette M. (2021). Epidemiology of Dermatophytes in Belgium: A 5 Years’ Survey. Mycopathologia.

[63] Martinez-Rossi N.M., Bitencourt T.A., Peres N.T.A., Lang E.A.S., Gomes E.V., Quaresemin N.R., Martins M.P., Lopes L., Rossi A. (2018). Dermatophyte Resistance to Antifungal Drugs: Mechanisms and Prospectus. Front. Microbiol..

[64] Saunte D.M.L., Hare R.K., Jørgensen K.M., Jørgensen R., Deleuran M., Zachariae C.O., Thomsen S.F., Bjørnskov-Halkier L., Kofoed K., Arendrup M.C. (2019). Emerging Terbinafine Resistance in *Trichophyton*: Clinical Characteristics, Squalene Epoxidase Gene Mutations, and a Reliable EUCAST Method for Detection. Antimicrob. Agents Chemother..

[65] Carmo P.H.F., Freitas G.J.C., Dornelas J.C.M., Almeida B.C.T., Baltazar L.M., Ferreira G.F., Peres N.T.A., Santos D.A. (2022). Reactive oxygen and nitrogen species are crucial for the antifungal activity of amorolfine and ciclopirox olamine against the dermatophyte *Trichophyton interdigitale*. Med. Mycol..

[66] Aris P., Wei Y., Mohamadzadeh M., Xia X. (2022). Griseofulvin: An Updated Overview of Old and Current Knowledge. Molecules.

[67] Gupta A.K., Mays R.R., Versteeg S.G., Piraccini B.M., Shear N.H., Piguet V., Tosti A., Friedlander S.F. (2018). *Tinea capitis* in children: A systematic review of management. J. Eur. Acad. Dermatol. Venereol..

[68] Branda F., Petrosillo N., Ceccarelli G., Giovanetti M., De Vito A., Madeddu G., Scarpa F., Ciccozzi M. (2025). Antifungal Agents in the 21st Century: Advances, Challenges, and Future Perspectives. Infect. Dis. Rep..

[69] Achilonu C.C., Davies A., Kanu O.O., Noel C.B., Oladele R. (2023). Recent Advances and Future Perspectives in Mitigating Invasive Antifungal-Resistant Pathogen *Aspergillus fumigatus* in Africa. Curr. Treat. Options Infect. Dis..

[70] Gupta A.K., Susmita Nguyen H.C., Liddy A., Economopoulos V., Wang T. (2025). Terbinafine Resistance in *Trichophyton rubrum* and *Trichophyton indotineae:* A Literature Review. Antibiotics.

[71] Kong X., Xie W., Fu M., Feng P., Li Z., Liu H., Tong Z., Abliz P., Jiang Y., Yang L. (2026). Antifungal resistance of the *Trichophyton mentagrophytes/Trichophyton interdigitale* species complex: Insights from the China Antifungal Resistance Dermatophytes Surveillance network Study (CARDS). J. Eur. Acad. Dermatol. Venereol..

[72] Gupta A.K., Cooper E.A., Wang T., Polla Ravi S., Lincoln S.A., Piguet V., McCarthy L.R., Bakotic W.L. (2023). Detection of Squalene Epoxidase Mutations in United States Patients with Onychomycosis: Implications for Management. J. Investig. Dermatol..

[73] Bhuiyan M.S.I., Verma S.B., Illigner G.M., Uhrlaß S., Klonowski E., Burmester A., Noor T., Nenoff P. (2024). *Trichophyton mentagrophytes* ITS Genotype VIII/*Trichophyton indotineae* Infection and Antifungal Resistance in Bangladesh. J. Fungi.

[74] Madarasingha N.P., Thabrew H., Uhrlass S., Eriyagama S., Reinal D., Jayasekera P.I., Nenoff P. (2024). Dermatophytosis Caused by *Trichophyton indotineae* (*Trichophyton mentagrophytes* ITS Genotype VIII) in Sri Lanka. Am. J. Trop. Med. Hyg..

[75] Astvad K.M.T., Hare R.K., Jørgensen K.M., Saunte D.M.L., Thomsen P.K., Arendrup M.C. (2022). Increasing Terbinafine Resistance in Danish Trichophyton Isolates 2019–2020. J. Fungi.

[76] McKinney W., Blakiston M., Roberts S., Morris A. (2025). Clinical alert: Arrival of terbinafine resistant *Trichophyton indotineae* in New Zealand. N. Z. Med. J..

[77] Bortoluzzi P., Prigitano A., Sechi A., Boneschi V., Germiniasi F., Esposto M.C., Romanò L., Pavan G., Matinato C., Veraldi S. (2023). Report of terbinafine resistant *Trichophyton* spp. in Italy: Clinical presentations, molecular identification, antifungal susceptibility testing and mutations in the squalene epoxidase gene. Mycoses.

[78] Liang T., Chen X., de Hoog G.S., Li L., Wang L., Wan Z., Yu J., Li R., Song Y. (2025). Antifungal Resistance Patterns of *Microsporum canis*: A 27-Year MIC Study in Mainland China. Mycoses.

[79] Núñez A., Silva V., Pereira M., Castro R. (2025). Antifungal susceptibility testing of *Microsporum canis* isolated from the skin of dermatologically healthy cats. Open Vet. J..

[80] Martinez-Rossi N.M., Peres N.T.A., Bitencourt T.A., Martins M.P., Rossi A. (2021). State-of-the-Art Dermatophyte Infections: Epidemiology Aspects, Pathophysiology, and Resistance Mechanisms. J. Fungi.

[81] Achilonu C.C., Kottom T.J., Limper A.H. (2026). Functional and molecular characterization of *Aspergillus fumigatus* phosphoglucomutase (Pgm): A potential target for antifungal therapy. J. Med. Microbiol..

[82] Yamada T., Maeda M., Alshahni M.M., Tanaka R., Yaguchi T., Bontems O., Salamin K., Fratti M., Monod M. (2017). Terbinafine Resistance of *Trichophyton* Clinical Isolates Caused by Specific Point Mutations in the Squalene Epoxidase Gene. Antimicrob. Agents Chemother..

[83] Santos H.L., Lang E.A.S., Segato F., Rossi A., Martinez-Rossi N.M. (2018). Terbinafine resistance conferred by multiple copies of the salicylate 1-monooxygenase gene in *Trichophyton rubrum*. Med. Mycol..

[84] Klinger M., Theiler M., Bosshard P.P. (2021). Epidemiological and clinical aspects of *Trichophyton mentagrophytes/Trichophyton interdigitale* infections in the Zurich area: A retrospective study using genotyping. J. Eur. Acad. Dermatol. Venereol..

[85] Abastabar M., Babaei M., Mohammadi R., Valadan R., Javidnia J., Zaedi A., Aghili S.R., Haghani I., Khojasteh S., Reazaei-Matehkolaei A. (2023). Iranian National Survey on *Tinea Capitis*: Antifungal Susceptibility Profile, Epidemiological Characteristics, and Report of Two Strains with a Novel Mutation in *SQLE* Gene with Homology Modeling. Mycopathologia.

[86] Kromer C., Celis D., Hipler U., Zampeli V.A., Mößner R., Lippert U. (2021). Dermatophyte infections in children compared to adults in Germany: A retrospective multicenter study in Germany. JDDG J. Dtsch. Dermatol. Ges..

[87] Selan M., Hrvatin Stančič B., Dolenc-Voljč M. (2023). *Tinea genitalis* profunda, a diagnostic challenge: A case report and literature review. Acta Dermatovenerol. Alp. Pannonica Adriat..

[88] Besrour R., Mtibaa L., Rabhi F., Baccouchi N., Dhaoui A., Jemli B. (2025). *Epidermophyton floccosum*, an etiological agent of *tinea pedis* and *tinea unguium*: About two cases. Pan Afr. Med. J..

[89] Yotsu R.R., Kouadio K., Yao A., Vagamon B., Takenaka M., Murota H., Makimura K., Nishimoto K. (2021). *Tinea Capitis* Caused by *Microsporum audouninii*: A Report of Two Cases from Côte D’Ivoire, West Africa. Trop. Med. Infect. Dis..

[90] Martínez Campayo N., Rego Campuzano I., González de Aledo M., Arévalo Bermúdez M.P., Fernández Torres R.M., Fonseca E. (2022). New Epidemiological Outcomes in Anthropophilic *tinea capitis*, a Case Series Study in Northwestern Spain. Actas Dermosifiliogr..

[91] Aragón-Sánchez J., López-Valverde M.E., Víquez-Molina G., Milagro-Beamonte A., Torres-Sopena L. (2023). Onychomycosis and *Tinea Pedis* in the Feet of Patients With Diabetes. Int. J. Low. Extrem. Wounds.

[92] Bruce Fraser R.D., Parry D.A.D. (2012). The role of disulfide bond formation in the structural transition observed in the intermediate filaments of developing hair. J. Struct. Biol..

[93] Gong H., Zhou H., McKenzie G.W., Yu Z., Clerens S., Dyer J.M., Plowman J.E., Wright M.W., Arora R., Bawden C.S. (2012). An Updated Nomenclature for Keratin-Associated Proteins (KAPs). Int. J. Biol. Sci..

[94] Bontems O., Fratti M., Salamin K., Guenova E., Monod M. (2020). Epidemiology of Dermatophytoses in Switzerland According to a Survey of Dermatophytes Isolated in Lausanne between 2001 and 2018. J. Fungi.

[95] Kupsch C., Czaika V., Deutsch C., Gräser Y. (2019). *Trichophyton mentagrophytes*—A new genotype of zoophilic dermatophyte causes sexually transmitted infections. JDDG J. Dtsch. Dermatol. Gesellschaft..

[96] Kermani F., Moosazadeh M., Hedayati M.T., Abastabar M., Haghani I., Aghili S.R., Shokohi T. (2020). Molecular epidemiology of *Tinea gladiatorum* in contact sports in northern Iran. Mycoses.

[97] Firooz A., Lotfali E., Fattahi M., Fattahi M., Miramin Mohammadi A., Shahrzad Kavkani M. (2021). A Case of Terbinafine-Resistant *Tinea Cruris* Caused by *Trichophyton tonsurans*. Case Rep. Dermatol. Med..

[98] Aimoldina A., Smagulova A., Batpenova G., Konnikov N., Algazina T., Jetpisbayeva Z., Azanbayeva D., Amantayev D., Kiyan V. (2025). Mycological Profile and Associated Factors Among Patients with Dermatophytosis in Astana, Kazakhstan. J. Fungi.

[99] Bitew A., Osman F., Yassin S. (2022). Non-Dermatophyte Mold Dominated Onychomycosis in Patients Attending a Rank Higher Specialized Dermatology Clinic in Addis Ababa, Ethiopia. Clin. Cosmet. Investig. Dermatol..

[100] Diawara E.Y., Keita A.K., Conde M., Kabtani J., Conde M., Diongue K., Ranque S. (2025). Dermatophytosis Among Schools Children in the Republic of Guinea.

[101] Agokeng D.A.J., Njateng G.S.S., Dabou S., Diongue K., Agokeng K.B.D., Ranque S. (2025). Prevalence and risk factors of *tinea capitis* in primary school children across four regions of Cameroon. New Microbes New Infect..

[102] Correia E.E.M., Mota M., Veiga LVAde M., Fernandes C., Gonçalves T. (2024). Predominance of *Trichophyton soudanense* as Agent of Dermatophytoses in Cape Verdean School-Age Children. J. Fungi.

[103] Chouaieb H., Akhoundi M., Fetoui N.G., Kalboussi Y., Ismail S., Khammari I., Brun S., Denguezli M., Fathallah A. (2025). *Tinea capitis* in adults: A 14-year retrospective study in central Tunisia. J. Med. Mycol..

[104] Araya S., Tesfaye B., Fente D. (2020). Epidemiology of Dermatophyte and Non-Dermatophyte Fungi Infection in Ethiopia. Clin. Cosmet. Investig. Dermatol..

[105] Zarzeka D., Benedict K., McCloskey M., Lockhart S.R., Lipner S.R., Gold J.A.W. (2024). Current epidemiology of *tinea corporis* and *tinea cruris* causative species: Analysis of data from a major commercial laboratory, United States. J. Am. Acad. Dermatol..

[106] Russo M.F., Almassio A., Abad M.E., Larralde M. (2024). *Tinea capitis* caused by *Trichophyton tonsurans*: An emerging disease in Argentina. Arch. Argent. Pediatría.

[107] González F.E., Rodríguez J.A., Muñoz L.M., Apráez G., Vásquez L.R. (2023). Outbreak of trichophytic *tinea capitis* among schoolchildren in a rural area of the Department of Cauca, Colombia. Biomédica.

[108] Brito S.C.P., Pinto M.R., Alcântara L.M., Reis N.F., Durães T.L., Bittar C.T.M., Oliveira J.C.d, Rocha E.M.d.S.d, Machado R.L.D., Guimarães R.J.d.P.S.E. (2023). Spatio-temporal six-year retrospective study on dermatophytosis in Rio de Janeiro, Southeast Brazil: A tropical tourist locality tale. PLoS Negl. Trop. Dis..

[109] Correia N.S., Balbinot R.T.S., Bonacorsi C., Donofrio F.C. (2022). Epidemiology of dermatomycoses in children in Northern Mato Grosso 2015–2020. Mycoses.

[110] Bonifaz A., Araiza J., Tirado-Sánchez A., Barbosa-Zamora A., Gómez-Sáenz A., Méndez-Juárez A. (2021). *Tinea gladiatorum* due to Trichophyton tonsurans in a school wrestling team in Mexico: A case series. Curr. Med. Mycol..

[111] Bitew A., Yilma B., Taye T. (2022). High *Trichophyton violaceum*-Induced *Tinea Capitis* with Isolation of Many Non-Dermatophyte Molds in Scalp Scrapings in Patients Referred to a Dermatology Clinic in Addis Ababa, Ethiopia. Clin. Cosmet. Investig. Dermatol..

[112] Gupta A.K., Thornbush M., Wang T. (2025). Climate Change, Natural Disasters, and Cutaneous Fungal Infections. Int. J. Dermatol..

[113] Salzer H.J.F., Stoney R.J., Angelo K.M., Rolling T., Grobusch M.P., Libman M., López-Vélez R., Duvignaud A., Ásgeirsson H., Crespillo-Andújar C. (2018). Epidemiological aspects of travel-related systemic endemic mycoses: A GeoSentinel analysis, 1997–2017. J. Travel Med..

[114] Ortiz B., Morio F., Aguilar K., García F., Fontecha G., Moreno-Sabater A. (2026). *T. indotineae*: A New Emergent Fungal Pathogen Driven by Global Travel. Mycoses.

[115] Kano R., Kimura U., Kakurai M., Hiruma J., Kamata H., Suga Y., Harada K. (2020). *Trichophyton indotineae* sp. nov.: A New Highly Terbinafine-Resistant Anthropophilic Dermatophyte Species. Mycopathologia.

[116] dos Santos A.R., Uhrlaß S., Nenoff P., Gold J.A.W., Bhuiyan M.S.I., Goturu S., Gade L., Bagal U.R., Peterson J.G., Wiederhold N.P. (2025). Global Emergence of Antifungal-Resistant Dermatophytosis Caused by *Trichophyton indotineae* (formerly *T. mentagrophytes ITS* Genotype VIII): A Genomic Investigation Involving 14 Countries. Mycoses.

[117] Jia S., Long X., Hu W., Zhu J., Jiang Y., Ahmed S., de Hoog G.S., Liu W., Jiang Y. (2023). The epidemic of the multiresistant dermatophyte *Trichophyton indotineae* has reached China. Front. Immunol..

[118] Al-Wathiqi F., Alharby A., Asadzadeh M., Alobaid K. (2025). A four-year retrospective study on epidemiological updates of dermatophytosis in Kuwait. BMC Infect. Dis..

[119] Moreno-Sabater A., Normand A.C., Bidaud A.L., Cremer G., Foulet F., Brun S., Bonnal C., Aït-Ammar N., Jabet A., Ayachi A. (2022). Terbinafine Resistance in Dermatophytes: A French Multicenter Prospective Study. J. Fungi.

[120] Jenks J.D., Prattes J., Wurster S., Sprute R., Seidel D., Oliverio M., Egger M., Del Rio C., Sati H., Cornely O.A. (2023). Social determinants of health as drivers of fungal disease. eClinicalMedicine.

[121] Pagano L., Fernández O.M. (2025). Clinical aspects and recent advances in fungal diseases impacting human health. J. Antimicrob. Chemother..

[122] Abdolrasouli A., Barton R.C., Borman A.M. (2025). Spread of Antifungal-Resistant *Trichophyton indotineae*, United Kingdom, 2017–2024. Emerg. Infect. Dis..

[123] Czerniewska A., Borman A., Abdolrasouli A., Budd E.L., Fisher M.C., Barton R., Bates K.A., Lambourne J., Demirjian A., Muller-Pebody B. (2025). Rapid emergence of *Trichophyton indotineae* (*Trichophyton mentagrophytes ITS* genotype VIII) observed in the United Kingdom, up to August 2025. Eurosurveillance.

[124] Mosam A., Shuping L., Naicker S., Maphanga T., Tsotetsi E., Mudau R., Maluleka C., Mpembe R., Ismail H., Singh A. (2023). A case of antifungal-resistant ringworm infection in KwaZulu-Natal Province, South Africa, caused by *Trichophyton indotineae*. Public Health Bull. S. Afr..

[125] Badiane A.S., Ramarozatovo L.S., Doumbo S.N., Dorkenoo A.M., Mandengue C., Dunaisk C.M., Ball M., Dia M.K., Ngaya G.S.L., Mahamat H.H. (2023). Diagnostic capacity for cutaneous fungal diseases in the African continent. Int. J. Dermatol..

[126] Chikoi R., Nyawale H.A., Mghanga F.P. (2018). Magnitude and Associated Risk Factors of Superficial Skin Fungal Infection Among Primary School Children in Southern Tanzania. Cureus.

[127] Amiri M., Furia F.F., Bakari M. (2020). Skin disorders among children living in orphanage centres in Dar es Salaam, Tanzania. Trop. Med. Health.

[128] Mushi M.F., Jonathan E., Mirambo M.M., Mshana S.E. (2019). Prevalence and Predictors of Dermatophyte Infections Among Primary School Children in Ilemela, Mwanza, Tanzania. E. Afr. Health Res. J..

[129] McTaggart L.R., Cronin K., Ruscica S., Patel S.N., Kus J.V. (2025). Emergence of terbinafine-resistant *Trichophyton indotineae* in Ontario, Canada, 2014–2023. J. Clin. Microbiol..

[130] Chua K.Y.L., Halliday C.L., Mason A., Vogrin S., Knox J., Chen S.C.A. (2025). Optimising the detection of *Trichophyton indotineae* and its prevalence in a large Australian laboratory. Pathology.

[131] Caplan A.S., Chaturvedi S., Zhu Y., Todd G.C., Yin L., Lopez A., Travis L., Smith D.J., Chiller T., Lockhart S.R. (2023). *Notes from the Field:* First Reported U.S. Cases of *Tinea* Caused by *Trichophyton indotineae*—New York City, December 2021–March 2023. MMWR Morb. Mortal. Wkly. Rep..

[132] Caplan A.S., Sikora M., Strome A., Akoh C.C., Otto C., Chaturvedi S., Zampella J.G. (2024). Potential Sexual Transmission of *Tinea Pubogenitalis* From TMVII. JAMA Dermatol..

[133] Cañete-Gibas C.F., Mele J., Patterson H.P., Sanders C.J., Ferrer D., Garcia V., Fan H., David M., Wiederhold N.P. (2023). Terbinafine-Resistant Dermatophytes and the Presence of *Trichophyton indotineae* in North America. J. Clin. Microbiol..

[134] de Almeida J.N., dos Santos A.R., Trindade MRde S., Gold J.A.W., Razo F.P.M., Gonçalves S.S., Dorlass E.G., Ruiz R.M., Pasternak J., Mangueira C.L.P. (2025). *Trichophyton indotineae* Infection, São Paulo, Brazil, 2024. Emerg. Infect. Dis..

[135] Messina F., Santiso G., Romero M., Bonifaz A., Fernandez M., Marin E. (2023). First case report of *tinea corporis* caused by *Trichophyton indotineae* in Latin America. Med. Mycol. Case Rep..

[136] Dellière S., Joannard B., Benderdouche M., Mingui A., Gits-Muselli M., Hamane S., Alanio A., Petit A., Gabison G., Bagot M. (2022). Emergence of Difficult-to-Treat *Tinea Corporis* Caused by *Trichophyton mentagrophytes* Complex Isolates, Paris, France. Emerg. Infect. Dis..

[137] Carrascal-Correa D.F., Zuluaga A., González A. (2020). Species distribution of the main aetiologic agents causing skin dermatophytosis in Colombian patients: A 23-year experience at a Mycological Reference Center. Mycoses.

[138] Orozco-Yee E.A., Rojas-Castañeda R.G., Guevara-Gutiérrez E., Mayorga-Rodríguez J., Tlacuilo-Parra A. (2025). Dermatophytosis caused by *Nannizzia gypsea*: Report of 155 cases in western Mexico. Enferm. Infecc. Microbiol. Clin..

[139] Ranorohasimanana N.M., Akhoundi M., Dorleans A., Benamari E., Rakotondrasoa S.R., Rasoavololona D.H., Razafindrakotosoa M.N., Izri A., Razanakolona L.R., Brun S. (2025). Prevalence of *tinea capitis* among schoolchildren in Mahajanga, northern Madagascar: An epidemio-clinical survey using conventional, proteomic and molecular approaches. J. Med. Mycol..

[140] Xie Z., Liu Y., Mo B., Chen Y., Lin E., Shen W., Xue Y., Yuan L., Liu H. (2025). Alert on imported fungal infection: The first report of *tinea capitis* due to *Microsporum audouinii* in China. Emerg. Microbes Infect..

[141] Zheng D., Liang T., Wu W., Al-Odaini N., Pan K., Huang L., Huang G., Tang L., Li X., He S. (2023). The Epidemiology of *Tinea Capitis* in Guangxi Province, China. Mycopathologia.

[142] Alshehri B.A., Alamri A.M., Rabaan A.A., Al-Tawfiq J.A. (2021). Epidemiology of Dermatophytes Isolated from Clinical Samples in a Hospital in Eastern Saudi Arabia: A 20-Year Survey. J. Epidemiol. Glob. Health.

[143] Wang X., Abuliezi R., Hasimu H., Zhang L., Abliz P. (2023). Retrospective Analysis of *Tinea Capitis* in Xinjiang, China. Mycopathologia.

[144] Agokeng D.A.J., Dabou S., Kabtani J., Agokeng K.B.D., Diongue K., Njateng G.S.S., Ranque S. (2024). Epidemiology of *Tinea Capitis* Among School-Children in Dschang, Western Cameroon. Mycopathologia.

[145] Farag A.G.A., Hammam M.A., Ibrahem R.A., Mahfouz R.Z., Elnaidany N.F., Qutubuddin M., Tolba R.R.E. (2018). Epidemiology of dermatophyte infections among school children in Menoufia Governorate, Egypt. Mycoses.

[146] Otašević S., Momčilović S., Golubović M., Ignjatović A., Rančić N., Đorđević M., Ranđelović M., Hay R., Arsić-Arsenijević V. (2019). Species distribution and epidemiological characteristics of superficial fungal infections in Southeastern Serbia. Mycoses.

[147] Pablo-Hernando E., Alcón J., Riesgo M., Benito R. (2025). Epidemiology of dermatophytes isolated from superficial dermatological samples taken during 2020–2023 in Zaragoza (Spain). Rev. Iberoam. Micol..

[148] Gupta A.K., Polla Ravi S., Wang T., Faour S., Bamimore M.A., Heath C.R., Friedlander S.F. (2024). An update on *tinea capitis* in children. Pediatr. Dermatol..

[149] Gits-Muselli M., Benderdouche M., Hamane S., Mingui A., Feuilhade de Chauvin M., Guigue N., Picat M., Bourrat E., Petit A., Bagot M. (2016). Continuous increase of *Trichophyton tonsurans* as a cause of *tinea capitis* in the urban area of Paris, France: A 5-year-long study. Med. Mycol..

[150] Gangneux J.P., Miossec C., Machouart M., Gits-Muselli M., Benderdouche M., Ranque S., Botterel F., Brun S., SFMM Tinea capitis Study Group (2024). Epidemiology and management of *tinea capitis* in France: A 6-year nationwide retrospective survey. Med. Mycol..

[151] Calander S., Saunte D., Polesie S. (2021). *Tinea Capitis* Caused by *Microsporum audouinii:* Lessons from a Swedish Community Outbreak. Acta Derm. Venereol..

[152] Johansen C.D., Shen J.J.R., Astvad K.M.T., Jemec G.B.E., Christensen J.J., Saunte D.M.L. (2024). Exploring treatment and antifungal resistance in an outbreak of *tinea* caused by *Microsporum audouinii*. Mycoses.

[153] Santino M.F.F., de Melo C.S., Melo A.S.A., Lima S.L., Paixão M.N., Akiti T., Barreiros G., Falcão E.M.M., Barbosa S.S. (2024). *Microsporum audouinii*: Emergence of an etiological agent of *tinea capitis* in Rio de Janeiro, Brazil (2012–2019). Med. Mycol..

[154] Sun P.L., Chi C.C., Shih I.H., Fan Y.C. (2023). *Nannizzia polymorpha* as Rare Cause of Skin Dermatophytosis. Emerg. Infect. Dis..

[155] Soankasina A.H., Rakotozandrindrainy N., Andrianteloasy S., Zafindraibe N.J., Rasamoelina T., Rafalimanana C., Cornet M., Razanakolona L.R., Rasamindrakotroka A., Andrianarivelo M.R. (2018). Dermatophyte infection caused by *Nannizzia gypsea:* A rare case report from Madagascar. Med. Mycol. Case Rep..

[156] Araya S., Abuye M., Negesso A.E. (2021). Epidemiological Characterization of Dermatomycosis in Ethiopia. Clin. Cosmet. Investig. Dermatol..

[157] Chen X.Q., Yu J. (2023). Global Demographic Characteristics and Pathogen Spectrum of *Tinea Capitis*. Mycopathologia.

[158] Mayser P., Nenoff P., Reinel D., Abeck D., Brasch J., Daeschlein G., Effendy I., Ginter-Hanselmayer G., Gräser Y., Hipler U. (2020). S1 guidelines: *Tinea capitis*. JDDG J. Dtsch. Dermatol. Gesellschaft..

[159] Dascalu J., Zaaroura H., Renert-Yuval Y., Khamaysi Z., Avitan-Hersh E., Friedland R. (2023). Pediatric *Tinea Capitis*: A Retrospective Cohort Study from 2010 to 2021. J. Fungi.

[160] Mirata D., Barberis C.S., Cerullo N., Ricci S., Silenzi F., Tronconi G., Filippeschi C., Oranges T. (2025). Epidemiology, diagnostic methods, and available treatments for *tinea capitis* in Ethiopia: A narrative review. Acta Trop..

